# Reconstitution of human CMG helicase ubiquitylation by CUL2^LRR1^ and multiple E2 enzymes

**DOI:** 10.1042/BCJ20210315

**Published:** 2021-07-23

**Authors:** Thanh Thi Le, Johanna Ainsworth, Cristian Polo Rivera, Thomas Macartney, Karim P.M. Labib

**Affiliations:** The MRC Protein Phosphorylation and Ubiquitylation Unit, School of Life Sciences, University of Dundee, Dundee DD1 5EH, U.K.

**Keywords:** CMG helicase, CUL2LRR1, cullin ligase, DNA synthesis and repair, p97, ubiquitins

## Abstract

Cullin ubiquitin ligases drive replisome disassembly during DNA replication termination. In worm, frog and mouse cells, CUL2^LRR1^ is required to ubiquitylate the MCM7 subunit of the CMG helicase. Here, we show that cullin ligases also drive CMG-MCM7 ubiquitylation in human cells, thereby making the helicase into a substrate for the p97 unfoldase. Using purified human proteins, including a panel of E2 ubiquitin-conjugating enzymes, we have reconstituted CMG helicase ubiquitylation, dependent upon neddylated CUL2^LRR1^. The reaction is highly specific to CMG-MCM7 and requires the LRR1 substrate targeting subunit, since replacement of LRR1 with the alternative CUL2 adaptor VHL switches ubiquitylation from CMG-MCM7 to HIF1. CUL2^LRR1^ firstly drives monoubiquitylation of CMG-MCM7 by the UBE2D class of E2 enzymes. Subsequently, CUL2^LRR1^ activates UBE2R1/R2 or UBE2G1/G2 to extend a single K48-linked ubiquitin chain on CMG-MCM7. Thereby, CUL2^LRR1^ converts CMG into a substrate for p97, which disassembles the ubiquitylated helicase during DNA replication termination.

## Introduction

Eukaryotic cells synthesise a single copy of their chromosomes in each cell cycle [1–3]. The control of chromosome duplication is achieved by the regulated assembly and disassembly of the CMG DNA helicase (CMG = CDC45_MCM2-7_GINS), which associates with a range of other factors at replication forks to form the eukaryotic replisome [4–9]. The assembly of the CMG helicase at origins of DNA replication is the key regulated step during the initiation of chromosome duplication, involving a mechanism that is broadly conserved in diverse eukaryotes [10–12]. CMG then remains stably associated with replication forks throughout elongation [13,14], until two replication forks converge, or a single fork reaches a DNA end, at which point CMG is disassembled and the replisome is thereby released from its DNA template [15–18].

The catalytic core of the CMG helicase comprises a hexameric ring of the six MCM2–7 ATPases [19], which is stabilised by association with the CDC45 protein and the GINS complex and encircles the template for leading strand DNA synthesis at replication forks [20]. In budding yeast and metazoa, disassembly of CMG during DNA replication termination is known to be induced by ubiquitylation of the CMG-MCM7 subunit [17,18], thereby driving recruitment of the Cdc48/p97 ATPase with its ubiquitin adaptors UFD1 and NPL4 [16,21,22]. Cdc48/p97 then unfolds ubiquitylated MCM7, irreversibly disrupting CMG into its component parts that are no longer able to associate with each other until the subsequent round of S-phase [16,23].

The ubiquitin ligases that induce CMG ubiquitylation have diverged considerably during the course of eukaryotic evolution. The best-characterised enzyme comes from the budding yeast *Saccharomyces cerevisiae*, in which the cullin 1 ligase SCF^Dia2^ drives CMG ubiquitylation during DNA replication termination [16,17,23,24]. Nevertheless, orthologues of SCF^Dia2^ are only found in yeasts. Subsequent work with metazoa indicated that a cullin 2 ligase called CUL2^LRR1^ is required to ubiquitylate CMG-MCM7 during DNA replication termination, in the early embryo of *Caenorhabditis elegans* [25], extracts of *Xenopus laevis* eggs [25,26] and also in mouse embryonic stem cells [27]. The LRR1 substrate receptor of CUL2^LRR1^ is essential for viability, both in *C. elegans* [28] and also in human cells [29–31]. Human CUL2^LRR1^ was shown to control cytoplasmic ubiquitylation of the p21 cell cycle regulator [32], together with degradation of the cytoskeleton-associated protein tyrosine phosphatase PTPN14 [33], but nuclear substrates of mammalian CUL2^LRR1^ are poorly characterised. Until now, the regulation of CMG disassembly in human cells had not been examined, and a direct role for CUL2^LRR1^ in CMG helicase ubiquitylation had yet to be confirmed *in vitro*.

Mechanistic studies of budding yeast SCF^Dia2^ have been driven by the reconstitution of CMG-Mcm7 ubiquitylation with purified proteins. In this way, recent work has shown that SCF^Dia2^ activates a single E2 ubiquitin-conjugating enzyme called Cdc34, which firstly primes and then elongates one or more K48-linked ubiquitin chains on CMG-Mcm7, thereby pushing Mcm7 beyond a ubiquitin threshold that governs the activity of Cdc48–Ufd1–Npl4 [16,23]. CUL2^LRR1^-dependent ubiquitylation of mammalian CMG-MCM7 had not previously been reconstituted *in vitro*. One complicating factor is the greater complexity of cullin ligase biology in metazoa, compared with yeasts. For example, work with human cullin ligases has shown that their activity is dependent upon neddylation of the cullin subunit, which induces structural changes in the ubiquitin ligase and activates the cognate E2 enzymes [34–36]. Correspondingly, a small-molecule inhibitor of the E1 enzyme for cullin neddylation blocks CMG helicase ubiquitylation in *Xenopus* egg extracts [25,26] and also in mouse embryonic stem cells [27], whereas cullin neddylation is not essential for CMG-Mcm7 ubiquitylation in budding yeast [23]. A further complicating factor in metazoa is that the ubiquitylation of cullin ligase substrates is dependent on multiple E2 ubiquitin-conjugating enzymes [37,38]. These have specialised roles, either in conjugating the first ubiquitin to the substrate (priming enzymes), or in the subsequent formation of a ubiquitin chain on the mono-ubiquitylated substrate (elongating enzymes).

Here, we explore the regulation of CMG ubiquitylation in human cells and report the *in vitro* reconstitution of human CMG-MCM7 ubiquitylation, involving neddylated CUL2^LRR1^ and multiple ubiquitin-conjugating enzymes. These findings confirm CMG-MCM7 as the first nuclear substrate of mammalian CUL2^LRR1^, misregulation of which has been linked to several human cancers [39–42].

## Results and discussion

### Regulation of CMG helicase disassembly in human cells by cullin ligase activity and the p97 unfoldase

**Figure 1.**
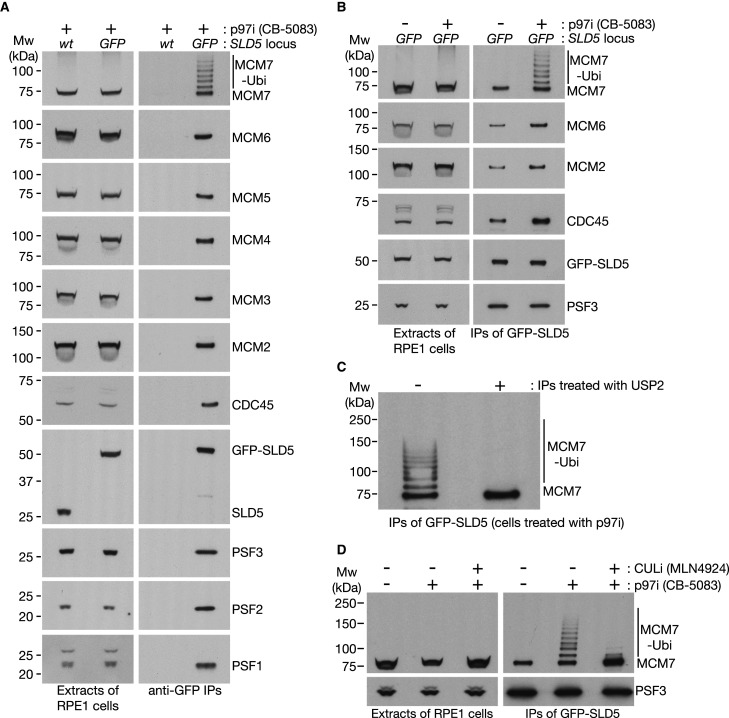
Regulation of CMG helicase disassembly in human RPE1 cells by cullin ligase activity and the p97 unfoldase. (**A,B**) Wild type and homozygous *GFP-SLD5* RPE1 cells were treated as indicated with 5 µM CB-5083 for 4 h. Cell extracts were incubated with anti-GFP beads and the indicated proteins monitored by immunoblotting. (**C**) Ubiquitylated CMG helicase was isolated by immunoprecipitation of GFP-SLD5 from cells treated with CB-5083 as above. The beads were then treated as indicated with 1 µM of human USP2 deubiquitylase, for 1 h at 24°C. (**D**) *GFP-SLD5* cells were treated as indicated with CB-5083 and 5 µM MLN4927 (inhibitor of cullin neddylation) for 6 h before harvesting and processing as above.

To isolate the CMG helicase from proliferating cells, we used CRISPR–Cas9 to modify both alleles of the *SLD5* gene in human RPE1 cells, in order to introduce green fluorescent protein (GFP) at the amino terminus of the SLD5 subunit of GINS (Supplementary Figure S1). Compared with mouse embryonic stem cells [27], isolating the endogenous CMG helicase from human cells is challenging, since cells spend less of the cell cycle in S-phase and the density of cells that can be grown in each culture dish is limited by contact inhibition. Nevertheless, by growing large cultures of *GFP-SLD5* cells, it was possible to isolate small amounts of human CMG (Figure 1A; see Materials and methods).

When cells were treated with a small-molecule inhibitor of the p97 unfoldase [43], CMG accumulated with ubiquitylated MCM7 (Figure 1B,C), uniquely amongst the eleven subunits of the helicase (Figure 1A). Moreover, the ubiquitylation of CMG-MCM7 was abrogated when cells were treated with the neddylation inhibitor MLN4927 in addition to p97 inhibitor (Figure 1D). These findings indicate that CMG-MCM7 ubiquitylation in the human cell cycle is dependent upon a cullin ubiquitin ligase, as observed recently in mouse embryonic stem cells [27] and previously in other metazoan species [25,26]. Ubiquitylated CMG is only detected upon inhibition of p97, likely reflecting the rapid disassembly of ubiquitylated CMG during DNA replication termination.

### CUL2^LRR1^ activates UBE2D enzymes to mono-ubiquitylate CMG-MCM7

As noted above, work with mouse ES cells has shown that CUL2^LRR1^ is required for the ubiquitylation of CMG-MCM7 during DNA replication termination, but mammalian CMG ubiquitylation had yet to be reconstituted *in vitro*, making it unclear whether the role of CUL2^LRR1^ was direct. We set out to address this issue for human CUL2^LRR1^, using a set of purified proteins (Supplementary Figure S2). Recent work with budding yeast proteins indicated that it is possible to reconstitute CMG helicase ubiquitylation in the absence of a DNA replication fork [16]. This is of physiological relevance, since replisome disassembly occurs not only when DNA replication terminates via the convergence of two replication forks [15,17,18], but also when a single replisome is released from a DNA end [14]. Correspondingly, recent work with *Xenopus* egg extracts has shown that the release of CMG from DNA replication forks is sufficient to induce CUL2^LRR1^-dependent ubiquitylation of CMG-MCM7 [44].

**Figure 2.**
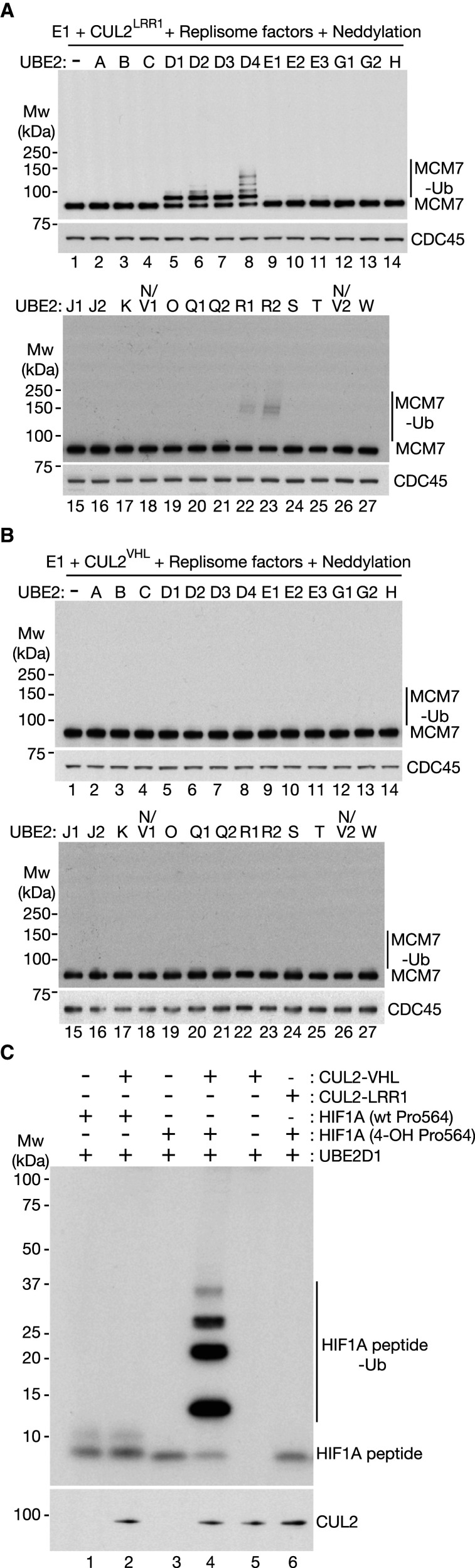
Human CUL2^LRR1^ activates UBE2D enzymes to prime ubiquitylation of CMG-MCM7. (**A**) Ubiquitylation reactions containing CUL2^LRR1^ and the indicated other components were performed as described in Materials and methods. Replisome factors were as shown in Supplementary Figure S2E, and ‘Neddylation’ indicates the presence of the factors shown in Supplementary Figure S2D. Ubiquitylation of CMG-MCM7 was monitored by immunoblotting. (**B**) Similar reactions were performed in the presence of CUL2^VHL^. (**C**) Ubiquitylation of HIF1A peptides containing unmodified or hydroxylated versions of Proline 564 were performed as described in Materials and methods, in the presence of the indicated factors. Ubiquitylation of the HIF1A peptides was monitored by immunoblotting.

To explore whether human CUL2^LRR1^ plays a direct role in CMG-MCM7 ubiquitylation in reconstituted reactions with purified proteins (Supplementary Figure S2), we monitored CMG-MCM7 ubiquitylation (Figure 2A) in the presence of UBA1 (E1 for ubiquitylation), a previously characterised panel of human E2 enzymes [45], CUL2^LRR1^, NEDD8 and its cognate E1/E2/E3 enzymes, and replisome components that are known to interact with the CMG helicase [9,46–48]. As a control, we performed equivalent reactions in which CUL2^LRR1^ was replaced with CUL2^VHL^ (Figure 2B), which mediates the ubiquitylation of the transcription factor HIF1 in response to hypoxia, dependent upon hydroxylation of HIF1 Proline 564 [49,50].

In reactions containing CUL2^VHL^, ubiquitylation of CMG-MCM7 was not seen with any of the tested E2 enzymes (Figure 2B). Nevertheless, CUL2^VHL^ induced the efficient and specific ubiquitylation of a HIF1 peptide containing a hydroxylated form of Proline 654 (Figure 2C), confirming the activity of the purified E3 enzyme. In contrast, CUL2^LRR1^ was unable to support HIF1 ubiquitylation (Figure 2C).

CMG-MCM7 ubiquitylation was not observed when CUL2^LRR1^ was combined with the majority of the tested E2 enzymes. However, approximately half of the CMG-MCM7 complexes were ubiquitylated in reactions combining CUL2^LRR1^ with members of the UBE2D family of E2 enzymes (Figure 2A, lanes 5–8). Mono-ubiquitylated CMG-MCM7 was the predominant product under the latter conditions, though UBE2D4 supported the conjugation of short ubiquitin chains to a fraction of CMG-MCM7 complexes (Figure 2A, lane 8; Supplementary Figure S3 shows that such chains were not formed in the presence of lysine-free ubiquitin, but were independent of lysine 48 of ubiquitin). A small proportion of CMG-MCM7 was also ubiquitylated in reactions containing CUL2^LRR1^ in combination with UBE2R1 or UBE2R2 (Figure 2A, lanes 22–23), consistent with previous work showing that UBE2R1/R2 are able, albeit very inefficiently, to prime the ubiquitylation of cullin substrates, in addition to carrying out ubiquitin chain extension [51]. These findings indicated that the CMG helicase is a specific and direct target of human CUL2^LRR1^, which principally activates the UBE2D family of E2 enzymes to mono-ubiquitylate CMG-MCM7.

### UBE2R and UBE2G extend ubiquitin chains on CMG-MCM7, dependent upon neddylated CUL2^LRR1^

**Figure 3.**
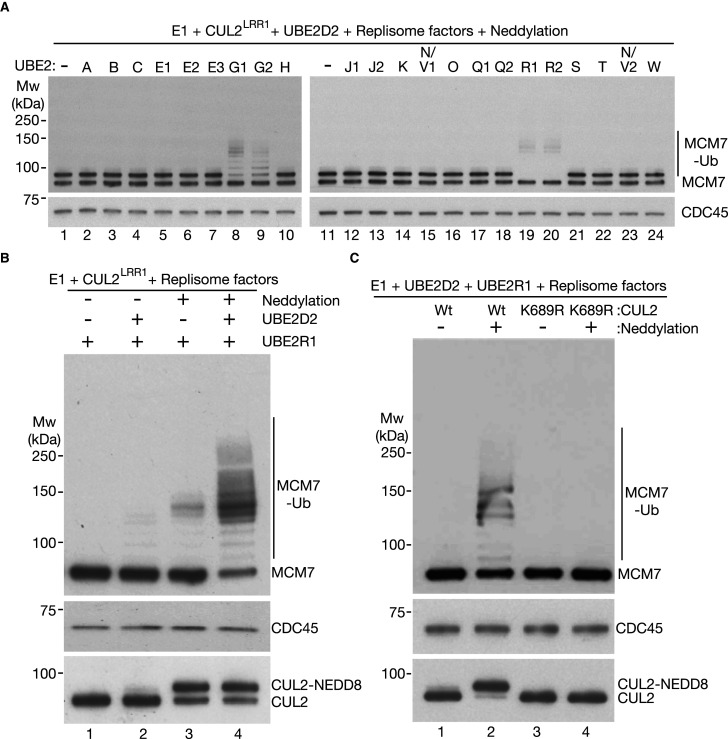
Neddylated CUL2^LRR1^ activates UBE2R1/R2 and UBE2G1/G2 to extend ubiquitin chains on CMG-MCM7 after priming by UBE2D. (**A**) Ubiquitylation reactions were performed as described above, except that all reactions contained UBE2D2, in addition to the indicated other E2 enzymes. ‘Neddylation factors’ indicates the presence of the factors shown in Supplementary Figure S2D and ‘Replisome factors’ are those shown in Supplementary Figure S2E. (**B**) The dependency of CMG-MCM7 ubiquitylation on neddylation was tested in the presence or absence of the indicated factors. (**C**) Similar reactions were performed in the presence of the indicated factors. CUL2-K689R has a mutation in the neddylation site that is important for cullin ligase activity.

During DNA replication termination, K48-linked polyubiquitylation of MCM7 converts the CMG helicase into a target for disassembly by the p97 unfoldase [16–18]. Past work indicated that UBE2R1/R2 are the principal E2 enzymes that are activated by human cullin E3 ligases to elongate ubiquitin chains [52]. Accordingly, we tested the panel of human E2 enzymes for ubiquitin chain formation and found that both CUL2^LRR1^ and CUL2^VHL^ predominantly activated UBE2R1/R2 to form K48-linked ubiquitin chains in the absence of substrate (Supplementary Figure S4A–C; UBE2D4 also supported a low level of free ubiquitin chain formation). To explore which E2 enzymes are able to extend K48-linked ubiquitin chains on human CMG-MCM7, we combined CUL2^LRR1^ and UBE2D2 with the remainder of the panel of E2 enzymes, in analogous reactions to those described above (UBE2D2 was chosen as a representative of UBE2D1–3 and was included to prime CMG-MCM7 ubiquitylation). With most of the tested E2 enzymes, the major product was mono-ubiquitylated CMG-MCM7, as seen when UBE2D2 was the only E2 enzyme in the reaction (Figure 3A, compare each lane with lane 1). In contrast, both UBE2R1 and UBE2R2 supported further ubiquitylation of CMG-MCM7 (Figure 3A, lanes 19–20), aided by UBE2D2 priming activity (compare with Figure 2, in which ubiquitylation of CMG-MCM7 was much less efficient in reactions containing only UBE2R). Strikingly, CUL2^LRR1^ also activated both UBE2G1 and UBE2G2 to extend ubiquitin chains on CMG-MCM7 in the presence of UBE2D2 (Figure 3A, lanes 8–9), These findings are consistent with previous studies that showed both *in vitro* and in human cells that UBE2D co-operates with UBE2R1/R2 or UBE2G1/G2 to ubiquitylate substrates of CUL4 and CUL1 and thereby induce substrate degradation [53–55]. Similarly, degradation of the CUL2^VHL^ substrate HIF1α in human cells was principally dependent on UBE2R1/R2, but degradation was further inhibited when UBE2G1/G2 were depleted in cells lacking UBE2R1/R2 [53].

In the combined presence of CUL2^LRR1^, UBE2D2, and UBE2R1/2 or UBE2G1/2, the major product of the reconstituted ubiquitylation reaction comprised CMG-MCM7 with ∼5–7 conjugated ubiquitin moieties (Figure 3A). Under such conditions, the observed ubiquitylation was almost entirely dependent upon neddylation of CUL2 on lysine 689 and was largely dependent upon priming of ubiquitylation by UBE2D2 (Figure 3B,C). These findings indicate that neddylated CUL2^LRR1^ firstly activates UBED2 enzymes to mono-ubiquitylate CMG-MCM7 and then activates either UBE2R1/R2 or UBE2G1/G2 to extend a polyubiquitin chain on CMG-MCM7.

### CUL2^LRR1^ specifically promotes the formation of K48-linked ubiquitin chains on the MCM7 subunit of CMG

**Figure 4.**
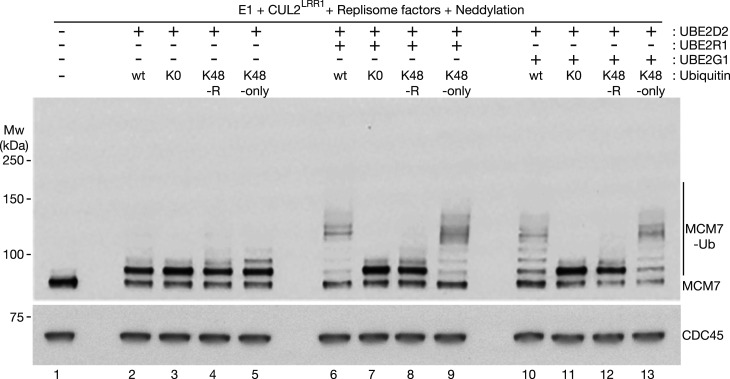
CUL2^LRR1^, UBE2D2 and UBE2R1/UBE2G1 co-operate to conjugate a single K48-linked ubiquitin chain to CMG-MCM7. Ubiquitylation reactions were performed as above, in the presence of the indicated factors. The reactions contained wild type ubiquitin (wt), lysine-free ubiquitin (K0), ubiquitin with lysine 48 mutated to arginine (K48R), or ubiquitin with all lysines mutated to arginine except for lysine 48 (K48-only). As above, ‘Neddylation’ indicates the presence of the factors shown in Supplementary Figure S2D and Replisome factors were as shown in Supplementary Figure S2E.

To explore the nature of the polyubiquitin chains that are conjugated to CMG-MCM7 in the presence of UBE2D2, UBE2R1, and UBE2G1, reactions with wild type ubiquitin were compared with reactions containing K0 ubiquitin, K48R ubiquitin or ubiquitin with all lysines mutated to arginine except lysine 48 (K48-only). With UBE2D2 as the only E2, ubiquitylation of CMG-MCM7 was comparable with all four versions of ubiquitin (Figure 4, lanes 2–5), thereby confirming that CUL2^LRR1^ activates UBE2D2 to mono-ubiquitylate CMG-MCM7.

We then combined CUL2^LRR1^ either with UBE2D2 and UBE2R1 (Figure 4, lanes 6–9), or with UBE2D2 and UBE2G1 (Figure 4, lanes 10–13). Whereas wild type ubiquitin and K48-only ubiquitin supported a similar pattern of polyubiquitylation of CMG-MCM7 (Figure 4, compare lanes 6, 9, 10 and 13), analogous reactions with either K0 or K48R ubiquitin led principally to monoubiquitylation of CMG-MCM7 (Figure 4, compare lanes 7, 8, 11 and 12). These findings indicate that both UBE2R1 and UBE2G1 extend a single K48-linked ubiquitin chain on CMG-MCM7 under these conditions. As described above, the majority of ubiquitylated CMG complexes receive a chain of at least five ubiquitin moieties under these conditions. Thereby, the ubiquitylated helicase is converted into a potential substrate for the p97/Cdc48 unfoldase, which has been shown to require a K48-linked chain of at least five ubiquitins [16,21,56].

**Figure 5.**
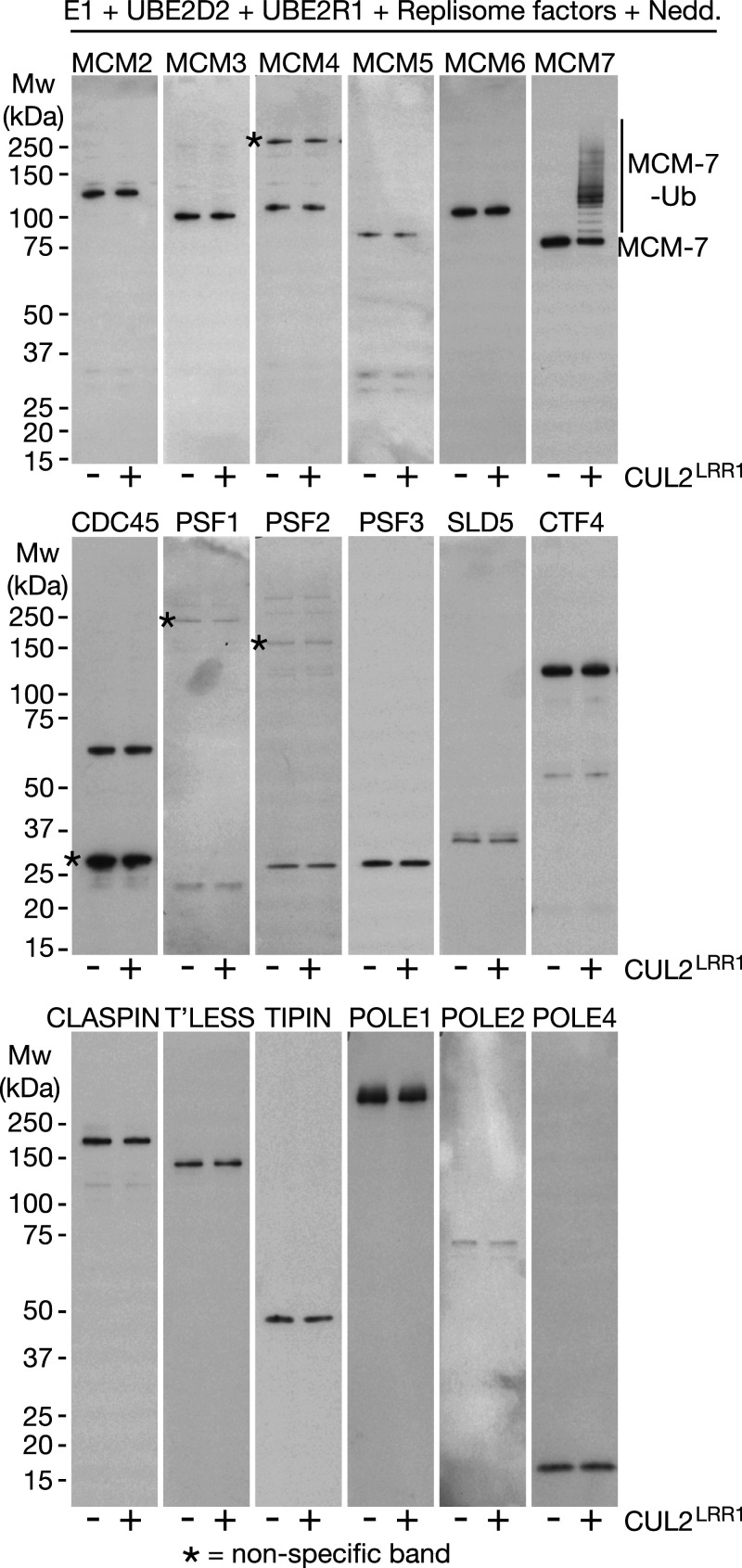
CUL2^LRR1^ is highly selective for CMG-MCM7 in the reconstituted ubiquitylation system. Reactions were performed as above, but in the presence or absence of CUL2^LRR1^ as shown. ‘Nedd.’ indicates the presence of the factors shown in Supplementary Figure S2D and ‘Replisome factors’ were as shown in Supplementary Figure S2E. Ubiquitylation of the indicated factors was monitored by immunoblotting. Asterisks indicate non-specific bands.

Finally, we examined the specificity of the reconstituted ubiquitylation reaction, using immunoblotting to monitor all eleven subunits of CMG, together with other replisome factors for which we had access to antibodies. As shown in Figure 5, the reaction was highly specific to MCM7, thereby recapitulating the high specificity of CMG helicase ubiquitylation that is observed in mammalian cells during DNA replication termination [27].

The chromosomal region encoding human LRR1, 14q21.3, is preferentially lost in metastatic lung adenocarcinoma, colorectal carcinoma and breast cancer [39–41], with loss of 14q21.3 also correlating with poor prognosis in squamous cell carcinomas of the head and neck [42]. The identification of CMG-MCM7 as a nuclear target of human CUL2^LRR1^ is an important step in exploring the potential links between LRR1 and tumour biology in future studies.

## Materials and methods

### Culture of human RPE1 cells

Human RPE1 cells were maintained in Dulbecco's modified Eagle medium : nutrion mixture F-12 (Thermo Fisher, DMEM/F-12, 21331020), supplemented with 10% foetal bovine serum (LabTech, FBS, FCS-SA/500), 2 mM l-Glutamine (Thermo Fisher, 25030081) and 100 U/ml Penicillin-Streptomycin (Thermo Fisher, 15140122) at 37°C in a humidified atmosphere of 5% CO_2_, 95% air. To release cells for passaging, plates were treated with 0.05% Trypsin/EDTA (Thermo Fisher, 25300054).

To inhibit the p97 unfoldase and allow time for cells to accumulate ubiquitylated CMG during DNA replication termination, we added 5 µM CB-5083 (Selleckchem, S8101) for 4 h before collection. To inhibit cullin ligase activity for the experiment in Figure 1D, before subsequently inactivating p97, we treated cells with 5 µM MLN4924 (Activebiochem, A-1139) for a total of 6 h before harvesting (2 h without CB-5083 plus 4 h in combination with CB-5083). MLN4924 inhibits the E1 enzyme for neddylation and thereby inactivates cullin ubiquitin ligases.

### CRISPR–Cas9 editing of human RPE1 cells

We used genome editing to alter both alleles of the endogenous *SLD5* locus, such that GFP was expressed as a fusion to the amino terminus of the SLD5 protein. A pair of guide RNAs (gRNAs) were designed for targeted cleavage of both parental DNA strands by the *S. pyogenes* Cas9 ‘nickase’ (Cas9-D10A), to generate two, off-set, single-stranded ‘nicks’ at the start codon of *SLD5*. Each of the two gRNAs contained 20 nucleotides of homology within exon 1 of human *SLD5*, which in the genomic locus is followed by a 3 nucleotide ‘protospacer adjacent motif’ (PAM) sequence that is required for cutting by Cas9 (as in Supplementary Figure S1A). The gRNA sequences were cloned as a pair of annealed oligonucleotides (TM16466 and TM16467 for gRNA1, TM16464 and TM16465 for gRNA2 — see Supplementary Table S1), into the vectors pX335 (Addgene Plasmid #42335) and pBABED P U6 (MRC PPU Reagents and Services, DU48788), using the Type IIS restriction enzyme BbsI, as described previously [57]. An additional guanine was added to the 5′ end of each gRNA sequence, to facilitate expression from the U6 promoter.

The resultant plasmids, expressing the pair of gRNAs along with Cas9-D10A and a puromycin resistance gene, were transfected into human RPE1 cells together with a plasmid containing ‘donor DNA’ (GFP flanked by ∼420–500 bp of homology to either side of the gRNA nicking sites within exon 1 of human *SLD5*). Sufficient silent mutations were introduced into the donor sequence within the gRNA target sites, centred on the PAM sequences, to prevent repeated nicking after successful insertion of the GFP tag (see Supplementary Figure S1B, lowercase text highlighted in red).

### Transfection of human RPE1 cells and selection of clones

One day prior to transfection, 0.2 × 10^6^ cells were plated per well of a 6-well dish in the supplemented DMEM/F-12 medium described above and incubated further. The next day, 1 µg of each plasmid DNA (two plasmids expressing the pair of gRNAs, Cas9 nickase and puromycin resistance, plus the donor vector containing GFP flanked by homology to human *SLD5* exon 1) was added to a 100 µl aliquot of DMEM/F-12 lacking supplements. In parallel, 10 µl Lipofectamine 2000 (Thermo Fisher, 11668030) was added to another 100 µl aliquot of 100 μl DMEM/F-12. The two aliquots were then mixed and incubated at room temperature for 5 min, before addition to a single well of pre-plated cells. For each transfection, reactions were prepared in two such wells. After an initial 24-h recovery periods, cells were grown for 24 h in fresh medium containing 5 µg/ml Puromycin (Thermo Fisher, A1113802), followed by 24 h of selection with fresh medium containing 2.5 µg/ml Puromycin. The surviving cells were then released from the well by the addition of 0.05% Trypsin/EDTA (Thermo Fisher, 25300054) and resuspended in DMEM/F-12 lacking supplements. GFP-positive cells were sorted by flow cytometry and individual cells were transferred to single wells of a 96-well plate, with each well containing 200 µl supplemented DMEM/F-12 medium. Colonies were observed ∼21 days later and viable clones were expanded, before analysis by immunoblotting, PCR and DNA sequencing, in order to screen for successful insertion of GFP into the human *SLD5* locus.

### PCR and sequence analysis to confirm GFP knockin to *SLD5* locus in human RPE1 cells

Cells from a 10-cm dish containing ∼3–4 × 10^6 ^cells were released with 0.05% Trypsin/EDTA as above, and 10% of the cells were then pelleted and resuspended in 50 µl of dH20. Subsequently, 50 µl of 100 mM NaOH was added with mixing, before heating at 95°C for 15 min. Following this incubation, 11 µl of 1 M Tris–HCl (pH 7.0) was added with mixing, and 1 µl was then used as the genomic DNA template in 25 µl PCR reactions with PrimeSTAR hot-start DNA polymerase (Takara Bio, R010B). The oligonucleotides used are outlined in Supplementary Table S1 and Supplementary Figure S1D.

### Preparation of human RPE1 cell extracts for immunoblotting and immunoprecipitation of protein complexes

Cells were seeded in 15-cm culture dishes at 8 × 10^5 ^cells per dish. Five such dishes were utilised for the experiments in Figure 1C,D and Supplementary Figure S1F. To provide sufficient material to monitor all 11 CMG subunits, the scale was increased seven-fold for the experiment in Figure 1A,B. For Supplementary Figure S1E, which only involved immunoblotting of cell extracts to monitor SLD5 and GFP-SLD5, rather than being used for immunoprecipitation of replication complexes, cells were grown as for normal maintenance and a single 10-cm dish with ∼3–4 × 10^6 ^cells was used to prepare an extract.

After growing as described above, the medium was removed and the cells were released from the dishes by incubation for 10 min in PBS containing 1 mM EGTA and 1 mM EDTA. The cell pellet was then collected by centrifugation and stored at −80°C. The typical yield was 0.18–0.25 g from five dishes grown as described above.

As described previously [27], cell pellets were thawed briefly on ice before resuspension 1 : 1(w/v) in lysis buffer (100 mM HEPES-KOH pH 7.9, 100 mM potassium acetate, 10 mM magnesium acetate, 2 mM EDTA, 10% glycerol, 0.1% Triton X-100, 2 mM sodium fluoride, 2 mM sodium β-glycerophosphate pentahydrate, 1 mM DTT, 1% Protease Inhibitor Cocktail (Sigma-Aldrich, P8215)). The activity of deubiquitylating enzymes was inhibited by the addition of 8 µM propargylated-ubiquitin (MRC PPU Reagents and Services, DU49003; [58]). Chromosomal DNA was then digested to release DNA-bound protein complexes, by the addition of 2500 U/ml of Pierce Universal Nuclease (Thermo Fisher, 88702). Samples were incubated at 4°C for 30 min with rotation. Subsequently, the samples were centrifuged at 20 000 ***g*** for 30 min at 4°C. A 50 μl aliquot of each supernatant was mixed with 50 μl of 1.5× Laemmli buffer, heated at 95°C for 5 min and then stored at −80°C for analysis.

The remainder of each supernatant was incubated with GFP-Trap agarose beads (Chromotek, gta-100) for 2 h at 4°C with rotation (5 μl bead slurry was used per 100 μl of recovered cell extract and the beads were first washed in 5 mg/ml BSA dissolved in PBS). Subsequently, the beads and associated proteins were washed four times with 1 ml pre-chillled ‘bead wash buffer’ (100 mM HEPES-KOH pH 7.9, 100 mM potassium acetate, 10 mM magnesium acetate, 0.1% IGEPAL CA-630), each pelleting the beads at 600 g for 1 min. Bound proteins were released by heating at 95°C for 5 min in 15–30 μl of 3× Laemmli buffer and stored at −80°C for subsequent analysis.

### Treatment of ubiquitylated CMG with human USP2 deubiquitylase

For the experiment in Figure 1C, cells were treated with 5 µM CB-5083 to inhibit CMG disassembly and promote CMG ubiquitylation. After immunoprecipitation of GFP-SLD5 and washing as above, the beads were resuspended in 100 µl bead wash buffer (100 mM HEPES-KOH pH 7.9, 100 mM potassium acetate, 10 mM magnesium acetate, 0.1% IGEPAL CA-630), supplemented with 1 µM human USP2 (MRC PPU Reagents and Services, DU13025). The samples were incubated at 24°C for 1 h, with agitation at 1400 rpm. The reactions were halted by removing the supernatant and adding 15 μl of 3× Laemmli buffer, before heating for 5 min at 95°C. Samples were stored at −80°C for subsequent analysis.

### Expression of E2 proteins in *E. coli* cells

The production of a panel of human E2 enzymes (Supplementary Table S1) was described previously [45]. In addition, human *UBE2R1*/*CDC34* was amplified from total RNA (QIAGEN, 338112) by RT-PCR and cloned by Gibson assembly into the vector pK27SUMO [59]. The resultant plasmid pTL2 (Supplementary Table S1) was transformed into *E. coli* Rosetta (DE3) pLysS (Novagen, 70956) and cells were then grown overnight at 37°C in 10 ml LB medium containing 50 µg/ml kanamycin, with shaking at 200 rpm in an INFORS HT Multitron Pro shaker (NR S-000119562-002). The following morning, the culture was diluted 100× into 1 l of selective medium. Once the cell density (OD_600_) had reached 0.8, a final concentration of 1 mM IPTG was added to induce the expression of 14His-SMT3-UBE2R1. Incubation was continued at 37°C for 4 h and cells were then harvested by centrifugation at 5000 rpm in a JLA-10.500 rotor (Avanti^TM^ J-25 centrifuge, Beckman) for 10 min at 4°C.

The cell pellet was washed once in cold PBS then resuspended in 20 ml buffer containing 30 mM HEPES KOH (pH 7.6), 10% Glycerol, 250 mM NaCl, 1 mM TCEP, 40 mM imidazole, Roche protease inhibitors (11873580001). For cell lysis, Lysozyme was added to a final concentration of 0.5 mg/ml. The mixture was incubated on ice for 20 min, followed by sonication at 40% amplitude, for three cycles of 30 s ON and 30 s OFF. The soluble fraction was recovered by centrifugation at 15 000 rpm in a JA-30.50 Ti fixed-angle titanium rotor (Avanti^TM^ J-25 centrifuge, Beckman) for 30 min at 4°C and then subjected to Ni^2+^ affinity purification by incubating with 2 ml of Ni-NTA resin (Qiagen, 30210), for 90 min at 4°C on a rotating wheel. The beads were recovered in a disposable gravity flow column and washed with 20 column volumes of wash buffer (30 mM HEPES KOH (pH 7.6), 10% Glycerol, 250 mM NaCl, 1 mM TCEP, 40 mM Imidazole) containing Roche protease inhibitors; then with 10 column volumes of wash buffer only.

The purified protein was eluted in buffer containing 30 mM HEPES KOH (pH 7.6), 10% Glycerol, 100 mM NaCl, 1 mM TCEP, 500 mM imidazole. The UBE2R1-containing fractions were pooled and the 14His-Smt3 tag was cleaved by incubation with Ulp1 protease [59]. The sample was concentrated to 0.5 ml using Amicon Ultra Centrifugal Filters (Millipore, UFC901024) before application to a Superdex 75 10/300 GL column purification in buffer containing 30 mM HEPES KOH (pH 7.6), 10% Glycerol, 100 mM NaCl, 1 mM TCEP. Peak fractions were pooled, concentrated by Amicon Ultra Centrifugal Filters (Millipore, UFC801024), snap frozen and stored at −80°C.

### Expression of human CMG in budding yeast cells

Codon optimised plasmids (Supplementary Table S1, pCPR1–6) to express the eleven subunits of human CMG from the bidirectional *GAL1*, *10* promoter in budding yeast were generated, using previously described methods [60]. Linearised versions of these plasmids were then transformed into the haploid yeast strains yJF1 and yCPR4 (Supplementary Table S1), which have opposite mating types and carry deletions in the genes expressing the Pep4 vacuolar protease and the Bar1 protease that degrades α-factor mating pheromone. The resultant strains were then mated to each other, producing a diploid strain that was sporulated, in order to isolate a haploid yeast strain (YCPR26, Supplementary Table S1) that expressed the 11 human CMG subunits.

### Purification of human CMG

Twelve-litre cultures of yeast cells (yCPR26) were grown at 30°C in YP medium supplemented with 2% Raffinose, until a cell density of 2.5–3 × 10^7 ^cells/ml was reached. The composition of YP medium is 1% (w/v) yeast extract (Becton Dickinson, 212750) and 2% (w/v) bacteriological peptone (Oxoid, LP0037B). Subsequently, galactose was added to a final concentration of 2% and incubation continued for 3 h to induce expression of the 11 components of human CMG. Cells were then harvested by centrifugation at 4000 rpm in a JLA-9.1000 rotor (Avanti^TM^ J-25 centrifuge, Beckman) for 10 min at 4°C. Cell pellets were washed once with ‘CMG buffer’ containing 25 mM HEPES KOH (pH 7.6), 10% Glycerol, 2 mM Mg(OAc)_2_, 300 mM KCl, 0.02% Tween-20 and 1 mM DTT. The washed cell pellets were then resuspended in 0.3 volumes of CMG buffer containing Roche protease inhibitors and 1 mM DTT. The cell suspension was frozen dropwise in liquid nitrogen to make ‘yeast popcorn’, as described previously [60]. Subsequently, frozen popcorn was ground in a freezer mill (SPEX CertiPrep 6850 Freezer/Mill) with 4 cycles of 2 min, at a rate of 15 cycles per second, in order to obtain yeast powder that was then stored at −80°C until required.

The frozen cell powder was thawed and resuspended with an equivalent volume of CMG buffer supplemented with protease inhibitors and the insoluble fraction was then removed by ultracentrifugation (45 000 rpm, 4°C, 1 h) in a type 45 Ti fixed-angle titanium rotor (Optima L-90K Ultracentrifuge, Beckman). The soluble protein extract was recovered in a disposable column and mixed with 5 ml of IgG Sepharose 6 Fast Flow beads (GE Healthcare, 17096901) pre-washed in CMG buffer and incubated for 2 h at 4°C on a rotating wheel. The beads were then recovered and washed extensively with 20–30 column volumes of CMG buffer lacking protease inhibitors, before the addition of 200 µg of TEV protease to allow tag cleavage during overnight incubation at 4°C with rotation.

Subsequently, the TEV eluate fraction was collected and the KCl concentration was adjusted to 200 mM using CMG buffer lacking KCl. The diluted fraction was loaded on a 0.24 ml MiniQ column (GE Healthcare, GE17-0686-01) pre-equilibrated in CMG buffer containing 200 mM KCl. The helicase was eluted in a 4 ml salt gradient from 200 to 600 mM KCl. Subsequently, CMG containing fractions were pooled and further purified by Superose 6 10/300 GL column (GE Healthcare, GE17-5172-01). The pooled Superose 6 fractions were then loaded again to a 0.24 ml MiniQ column for protein concentration. Finally, the sample was dialysed against buffer containing 25 mM HEPES KOH (pH 7.6), 10% Glycerol, 2 mM Mg(OAc)_2_, 250 mM KOAc, 0.02% Tween-20, 1 mM DTT. Aliquots were snap frozen and stored at −80°C.

### Recombinant DNA construction for baculovirus expression

UBA1, CUL2^LRR1^, CUL2^VHL^, CTF4, CLASPIN, TIMELESS_TIPIN, POLε were expressed in insect cells, using the biGBac system for baculoviral expression as previously described [61]. Briefly, genes of interest were amplified from human total RNA (QIAGEN) by RT-PCR, or synthesised (Genscript), before cloning into pLIB vector [61] by Gibson assembly. For multiple subunit proteins, gene expression cassettes (GEC) were grouped into pBIG1 vectors. Finally, donor plasmids (pLIB-GEC or pBIG1s-GECs) were transformed into *E. coli* DH10Bac (Gibco, 10361012) to generate bacmids. Details of tagging information and plasmids used for each protein expression are shown in Supplementary Table S1.

### Baculovirus infection of insect cells

Five millilitre of Sf9 cells (0.9 × 10^6 ^cells/ml) in Sf-900 II SFM medium (Gibco, 10902104) were seeded into a T-25 flask and incubated for 30 min for cell attachment. Meanwhile, a transfection mixture was prepared by incubating 2 µg bacmid with 10 µl of Cellfectin II reagent (Gibco, 10362100) in 2 ml of Sf-900 II SFM medium for 30 min. Subsequently, adherent cells were incubated overnight with the transfection mixture at 27°C. The following morning, cells were washed once with 5 ml of Sf-900 II SFM medium containing ‘antibiotic-antimycotic’ (Gibco, 15240096) and then resuspended with 5 ml of Sf-900 II SFM medium containing ‘antibiotic-antimycotic’. The adherent cell culture was then incubated at 27°C for 5 days. The resultant ‘P1 viral stock’ was isolated by taking the supernatant after centrifugation at 1500 ***g*** for 5 min at room temperature. The viral suspension was supplemented with 2% FBS before storage at 4°C, in the dark.

### Baculovirus amplification in insect cells

Sf21 cells were added to a final concentration of 1.5 × 10^6 ^cells/ml in 300 ml of Sf-900 II SFM medium containing ‘antibiotic-antimycotic’, and P1 viral stock was added to a final concentration of 1%. The suspension culture was incubated at 27°C with shaking at 110 rpm in an INFORS HT Multitron Pro shaker (NR S-000119562-002) for 4 days. The resultant ‘P2 viral stock’ was then recovered by centrifugation at 1500 ***g*** for 10 min at room temperature. FBS was added to a final concentration of 2%, before storage of the amplified viruses at 4°C, in the dark. If necessary, the above procedure was repeated to generate a more concentrated ‘P3 viral stock’.

### Protein expression and purification from baculovirus-infected insect cells

#### UBA1

A P2 viral stock for Flag-Tev-UBA1 was added to a 1 l culture of Sf21 cells (1.5 × 10^6^ cells/ml) to a final concentration of 10%. The suspension culture was incubated for 3 days at 27°C, with 110 rpm shaking in an INFORS HT Multitron Pro shaker (NR S-000119562-002). The cells were harvested by centrifugation at 1500 ***g*** for 10 min at 4°C. The cell pellet was then washed once with 1× cold PBS and resuspended in 50 ml buffer containing 25 mM HEPES KOH (pH 7.6), 10% Glycerol, 500 mM NaCl, 1 mM TCEP, Roche protease inhibitors, 1% Triton X-100; followed by 20 min incubation on a roller mixer at 4°C. The soluble fraction was recovered by centrifugation at 45 000 rpm, for 1 h at 4°C in a type 45 Ti fixed-angle titanium rotor (Optima L-90K Ultracentrifuge, Beckman), before subsequent incubation with 1.5 ml of anti-Flag M2 resin (Sigma–Aldrich, A2220) for 90 min at 4°C on a rotating wheel. The beads were recovered in a disposable gravity flow column and washed with 20 column volumes of wash buffer (25 mM HEPES KOH (pH 7.6), 10% Glycerol, 500 mM NaCl, 1 mM TCEP) containing Roche protease inhibitors; then with 15 column volumes of wash buffer. The protein was eluted in buffer containing 25 mM HEPES KOH (pH 7.6), 10% Glycerol, 200 mM NaCl, 1 mM TCEP, 0.5 mg/ml 3XFLAG peptide (Sigma–Aldrich, F4799). The UBA1-containing fractions were then pooled. The tag was removed by TEV cleavage and a further purification step was carried out with a Superdex 200 10/300 GL column in buffer containing 25 mM HEPES KOH (pH 7.6), 10% Glycerol, 200 mM NaCl, 1 mM TCEP. The peak fractions were pooled, concentrated using Amicon Ultra Centrifugal Filters (Millipore, UFC803024), snap frozen and stored at −80°C.

#### *CUL2^LRR1^*(containing wt CUL2 or CUL2-K689R)

The pentameric complex was expressed in a 1 l suspension culture containing 1.5 × 10^6^ cells/ml Sf21, by mixing with 5% ‘P3 viral stock’ harbouring CUL2_RBX1 (or CUL2-K689R_RBX1) plus 10% ‘P2 viral stock’ harbouring LRR1_Streptag-Tev-ELOB_ELOC. The suspension of cells and viruses was then incubated for 2.5 days at 27°C with shaking at 110 rpm in an INFORS HT Multitron Pro shaker (NR S-000119562-002). Cells were harvested by centrifugation at 1500 ***g*** for 10 min at 4°C and the cell pellet was washed once with 1× cold PBS, before resuspension in 50 ml buffer containing 25 mM HEPES KOH (pH 7.6), 10% Glycerol, 500 mM NaCl, 1 mM TCEP, 0.02% NP-40, plus Roche protease inhibitors. Cells were then lysed by sonication at 10% amplitude with 4 cycles of 30 s ON and 30 s OFF. The insoluble fraction was separated by 45 000 rpm centrifugation at 4°C for 1 h in a type 45 Ti fixed-angle titanium rotor (Optima L-90K Ultracentrifuge, Beckman). The supernatant was incubated with 2 ml of Strep-Tactin Superflow beads (IBA Lifesciences, 2-1206-025) on a rotating wheel for 90 min at 4°C. The beads were recovered and washed extensively with 20 column volumes of wash buffer (25 mM HEPES KOH (pH 7.6), 10% Glycerol, 500 mM NaCl, 1 mM TCEP, 0.02% NP-40) containing Roche protease inhibitors, then 20 column volumes of wash buffer containing 10 mM Mg(OAc)_2_, 2 mM ATP and finally 20 column volumes of wash buffer. Elution was performed in buffer containing 25 mM HEPES KOH (pH 7.6), 10% Glycerol, 500 mM NaCl, 1 mM TCEP, 0.02% NP-40, 7 mM d-Desthiobiotin. Peak fractions were pooled, concentrated to 0.5 ml and subjected to Superdex 200 10/300 GL column purification in buffer containing 25 mM HEPES KOH (pH 7.6), 10% Glycerol, 500 mM NaCl, 1 mM TCEP, 0.02% NP-40. Peak fractions were pooled and dialysed against buffer containing 25 mM HEPES KOH (pH 7.6), 10% Glycerol, 500 mM KOAc, 1 mM TCEP, 0.02% NP-40, then concentrated using Amicon Ultra Centrifugal Filters (Millipore, UFC803024). Finally, purified protein samples were aliquoted and snap frozen before storage at −80°C.

#### CUL2^VHL^

A 1 l culture of Sf21 cells at 1.5 × 10^6 ^cells/ml in Sf-900 II SFM medium containing ‘antibiotic-antimycotic’ was supplemented with 7% P3 viral stock for CUL2_RBX1 and 3.5% P3 viral stock for VHL_Streptag-Tev-ELOB_ELOC. The suspension of cells and viruses was then incubated for 2.5 days at 27°C with shaking at 110 rpm in an INFORS HT Multitron Pro shaker (NR S-000119562-002). Cells were harvested by centrifugation at 1500 ***g*** for 10 min at 4°C and the cell pellet was washed once with 1× cold PBS, before resuspension in 50 ml buffer containing 25 mM HEPES KOH (pH 7.6), 10% Glycerol, 350 mM NaCl, 1 mM TCEP, 0.02% NP-40, Roche protease inhibitors. Cells were then lysed by sonication at 10% amplitude with 4 cycles of 30 s ON and 30 s OFF. The supernatant was recovered by centrifuging at 45 000 rpm, 4°C for 1 h in a type 45 Ti fixed-angle titanium rotor (Optima L-90K Ultracentrifuge, Beckman) and subsequently subjected to Strep-Tactin affinity purification by adding 2 ml of Strep-Tactin Superflow beads (IBA Lifesciences, 2-1206-025). The mixture was incubated at 4°C on a rotating wheel for 90 min. The beads were then recovered and washed extensively with 20 column volumes of wash buffer (25 mM HEPES KOH (pH 7.6), 10% Glycerol, 350 mM NaCl, 1 mM TCEP, 0.02% NP-40) containing Roche protease inhibitors, followed by 20 column volumes of wash buffer containing 10 mM Mg(OAc)_2_, 2 mM ATP and finally 20 column volumes of wash buffer. Elution was performed in buffer containing 25 mM HEPES KOH pH 7.6, 10% Glycerol, 350 mM NaCl, 1 mM TCEP, 0.02% NP-40, 7 mM d-Desthiobiotin. Peak fractions were pooled, concentrated to 0.5 ml and applied to a Superdex 200 10/300 GL column that was equilibrated with buffer containing 25 mM HEPES KOH (pH 7.6), 10% Glycerol, 350 mM NaCl, 1 mM TCEP, 0.02% NP-40. Peak fractions were pooled, concentrated by Amicon Ultra Centrifugal Filters (Millipore, UFC803024) and aliquoted, before snap freezing and storage at −80°C.

#### CTF4

A final concentration of 5% P3 viral stock harbouring Streptag-Tev-CTF4 was added to a 2 l culture of Sf21 cells (1.5 × 10^6 ^cells/ml) in Sf-900 II SFM medium containing ‘antibiotic-antimycotic’. The suspension culture was incubated at 27°C with 110 rpm shaking for 2.5 days in an INFORS HT Multitron Pro shaker (NR S-000119562-002). Cells were then harvested by centrifugation at 1500 ***g*** for 10 min at 4°C. After washing once with 1× cold PBS, the cell pellet was resuspended in 100 ml buffer containing 25 mM HEPES KOH (pH 7.6), 10% Glycerol, 450 mM NaCl, 1 mM TCEP, 0.02% NP-40, Roche protease inhibitors. Cells were then lysed by sonication at 10% amplitude with 4 cycles of 30 s ON and 30 s OFF. The soluble material was recovered by centrifugation at 45 000 rpm at 4°C for 1 h in a type 45 Ti fixed-angle titanium rotor (Optima L-90K Ultracentrifuge, Beckman) and subsequently subjected to affinity purification by incubation with 3 ml of Strep-Tactin Superflow beads for 90 min at 4°C on a rotating wheel. The beads were recovered and washed extensively with 20 column volumes of wash buffer (25 mM HEPES KOH (pH 7.6), 10% Glycerol, 450 mM NaCl, 1 mM TCEP, 0.02% NP-40) containing Roche protease inhibitors, then with 10 column volumes of wash buffer. Elution was performed with buffer containing 25 mM HEPES KOH (pH 7.6), 10% Glycerol, 450 mM NaCl, 1 mM TCEP, 0.02% NP-40, 7 mM d-Desthiobiotin, before cleavage of the tag with Tev protease and further purification on a Superose 6 10/300 GL column, in buffer containing 25 mM HEPES KOH (pH 7.6), 10% Glycerol, 500 mM KOAc, 1 mM TCEP, 0.02% NP-40. Peak fractions were pooled, concentrated by Amicon Ultra Centrifugal Filters (Millipore, UFC803024), aliquoted, snap frozen and stored at −80°C.

#### CLASPIN

An expression culture was made by supplementing 5% P2 viral stock for Streptag-Tev-CLASPIN to a 1 l culture of Sf21 cells at a final concentration of 1.5 × 10^6 ^cells/ml, in Sf-900 II SFM medium containing ‘antibiotic-antimycotic’. After incubation for 2 days at 27°C with shaking at 110 rpm in an INFORS HT Multitron Pro shaker (NR S-000119562-002), cells were harvested by centrifugation at 1500 ***g*** for 10 min at 4°C and then washed once with 1× cold PBS. The cell pellet was then resuspended in 50 ml buffer containing 25 mM HEPES KOH (pH 7.6), 10% Glycerol, 300 mM NaCl, 1 mM TCEP, 0.1% Triton X-100, Roche protease inhibitors, Sigma protease inhibitors. Cells were then lysed by sonication at 10% amplitude with 4 cycles of 30 s ON and 30 s OFF. The insoluble fraction was removed by centrifugation at 45 000 rpm at 4°C for 1 h in a type 45 Ti fixed-angle titanium rotor (Optima L-90K Ultracentrifuge, Beckman). The soluble fraction was incubated with 2 ml of Strep-Tactin Superflow beads at 4°C on a rotating wheel for 90 min. The beads were recovered in a disposable gravity flow column and washed extensively with 20 column volumes of wash buffer (25 mM HEPES KOH (pH 7.6), 10% Glycerol, 300 mM NaCl, 1 mM TCEP) containing Roche protease inhibitors, Sigma protease inhibitors, followed by 20 column volumes of wash buffer. Elution was then performed in buffer containing 25 mM HEPES KOH (pH 7.6), 10% Glycerol, 300 mM NaCl, 1 mM TCEP, 7 mM d-Desthiobiotin. Peak fractions were pooled, incubated with Tev protease for 4 h for tag cleavage and applied to a HiTrap Q column, before elution with a gradient of 0.2–1 M NaCl. The pool of peak fractions was dialysed against storage buffer (25 mM HEPES KOH (pH 7.6), 10% Glycerol, 250 mM KOAc, 1 mM TCEP), before concentration with Amicon Ultra Centrifugal Filters (Millipore, UFC803024). Samples were then aliquoted, snap frozen and stored at −80°C.

#### TIMELESS-TIPIN

A 1 l culture of Sf21 cells (1.5 × 10^6 ^cells/ml) was mixed with 5% P2 viral stock harbouring Streptag-Tev-TIMELESS and 5% P2 viral stock for TIPIN. The mixture was then incubated at 27°C for two days with 110 rpm shaking in an INFORS HT Multitron Pro shaker (NR S-000119562-002). Cells were then harvested by centrifugation at 1500 ***g*** for 10 min at 4°C. The cell pellet was washed once with 1X cold PBS and resuspended in 50 ml buffer containing 25 mM HEPES KOH (pH 7.6), 10% Glycerol, 150 mM NaCl, 1 mM TCEP, 0.1% Triton X-100, Roche protease inhibitors. Cells were then lysed by sonication at 10% amplitude with 4 cycles of 30 s ON and 30 s OFF. The supernatant was recovered by centrifugation at 45 000 rpm at 4°C for 1 h in a type 45 Ti fixed-angle titanium rotor (Optima L-90K Ultracentrifuge, Beckman) and supplemented with 2 ml of Strep-Tactin Superflow beads. The mixture was left on a rotating wheel for 90 min at 4°C, before removal of the flow through with a disposable gravity flow column. The resin was then washed extensively with 20 column volumes of wash buffer (25 mM HEPES KOH (pH 7.6), 10% Glycerol, 150 mM NaCl, 1 mM TCEP, 0.02% NP-40) containing Roche protease inhibitors, followed by 20 column volumes of wash buffer. Elution was performed with buffer containing 25 mM HEPES KOH (pH 7.6), 10% Glycerol, 150 mM NaCl, 1 mM TCEP, 0.02% NP-40, 7 mM d-Desthiobiotin. The tag was cleaved out by incubating the eluted fractions with Tev protease at 4°C on a rotating wheel overnight. The following morning, the sample was concentrated to 0.5 ml and loaded onto a Superdex 200 10/300 GL column. Peak fractions were pooled, dialysed against storage buffer (25 mM HEPES KOH (pH 7.6), 10% Glycerol, 250 mM KOAc, 1 mM TCEP, 0.02% NP-40), concentrated by Amicon Ultra Centrifugal Filters (Millipore, UFC803024), aliquoted, snap frozen and stored at −80°C.

#### DNA polymerase epsilon

A 3 l suspension culture of Sf21 cells (1.5 × 10^6 ^cells/ml) was mixed with 10% P3 viral stock harbouring POLE1, and 1% P3 viral stock harbouring POLE2_Streptag-Tev-POLE3_POLE4. The mixture was incubated at 27°C with shaking at 110 rpm for 2 days in an INFORS HT Multitron Pro shaker (NR S-000119562-002). The cells were then harvested by centrifugation at 1500 ***g*** for 10 min at 4°C. After washing once with 1× cold PBS, the cell pellet was resuspended in 150 ml buffer containing 25 mM HEPES KOH (pH 7.6), 10% Glycerol, 500 mM KOAc, 1 mM TCEP, 0.1% Triton X-100, Roche protease inhibitors. Cells were then lysed by sonication at 10% amplitude with 4 cycles of 30 s ON and 30 s OFF. The insoluble fraction was removed by centrifugation at 45 000 rpm at 4°C for 1 h in a type 45 Ti fixed-angle titanium rotor (Optima L-90K Ultracentrifuge, Beckman). The supernatant was then incubated with 2 ml of Strep-Tactin Superflow beads at 4°C for 90 min on a rotating wheel. The beads were recovered in a disposable gravity flow column and washed extensively with 20 column volumes of wash buffer (25 mM HEPES KOH (pH 7.6), 10% Glycerol, 500 mM KOAc, 1 mM TCEP) containing Roche protease inhibitors, followed by 20 column volumes of wash buffer only. Elution was performed in buffer containing 25 mM HEPES KOH (pH 7.6), 10% Glycerol, 500 mM KOAc, 1 mM TCEP, 7 mM d-Desthiobiotin. The sample was then further purified on a Superdex 200 10/300 GL column in buffer containing 25 mM HEPES KOH (pH 7.6), 10% Glycerol, 500 mM KOAc, 1 mM TCEP. Peak fractions were then pooled and concentrated using Amicon Ultra Centrifugal Filters (Millipore, UFC803024), before aliquoting, snap freezing and storage at −80°C.

### *In vitro* CMG ubiquitylation assays

Reactions were assembled on ice in buffer containing 25 mM HEPES KOH (pH 7.6), 75 mM KOAc, 10 mM Mg(OAc)_2_, 1 mM DTT, 0.1 mg/ml BSA, 0.02% NP-40, 5 mM ATP, 6 µM ubiquitin, typically in a 10 µl final volume. The reactions contained 30 nM UBA1, UBE2R1 (100 nM in Figures 3B,C, 4, and 5 or 200 nM in other figures), 200 nM other E2 enzymes where indicated, 50 nM CUL2^LRR1^ or 50 nM CUL2^VHL^ as indicated, neddylation machinery as indicated (30 nM APP-BP1_UBA3, 30 nM UBE2M, 30 nM DCUN1D1) and replisome components as indicated (15 nM CMG, 30 nM CTF4, 45 nM CLASPIN, 45 nM TIMELESS_TIPIN, 30 nM POLε). The reactions were incubated at 30°C for 30 min and then stopped by the addition of SDS–PAGE sample loading buffer, before heating at 95°C for 5 min.

### HIF1 ubiquitylation assay

10 µl reactions containing the neddylation machinery (30 nM APP-BP1_UBA3, 30 nM UBE2M, 30 nM DCUN1D1), 30 nM UBA1, 200 nM UBE2D1, 50 nM CUL2^LRR1^ or 50 nM CUL2^VHL^ as indicated and 1 µM HIF1A peptide substrate were assembled on ice in buffer containing 25 mM HEPES KOH (pH 7.6), 75 mM KOAc, 10 mM Mg(OAc)_2_, 1 mM DTT, 0.1 mg/ml BSA, 0.02% NP-40, 5 mM ATP, 6 µM ubiquitin. Reactions were incubated at 30°C for 30 min and then stopped by the addition of SDS–PAGE sample loading buffer, before heating at 95°C for 5 min.

### Immunoblotting

Protein samples were resolved by SDS–PAGE on NuPAGE Novex 4–12% Bis-Tris gels (ThermoFisher Scientific) with NuPAGE MOPS SDS Running buffer (ThermoFisher Scientific), or with NuPAGE MES SDS Running buffer (ThermoFisher Scientific) to improve resolution when monitoring unattached ubiquitin chains (Supplementary Figure S4) or ubiquitylated HIF1A peptide (Figure 2C). The resolved proteins were transferred onto a nitrocellulose iBlot membrane (Invitrogen, IB301031) with the iBlot Dry Transfer System (Invitrogen, Serial No.10063176). Immunoblotting was performed with the antibodies shown in Supplementary Table S1. Detection was carried out with the ECL Western Blotting Detection Reagent kit (GE Healthcare, RPN 2106). The chemiluminescent signal was captured on Hyperfilm ECL film (Amersham, GE Healthcare, 28906837) and developed with an ECOMAX X-Ray Film Processor (Protec).

## Data Availability

There are no large data sets associated with this manuscript. Reagents described in this study are available from MRC PPU Reagents and Services (https://mrcppureagents.dundee.ac.uk).

## Abbreviations

GEC: gene expression cassettes
GFP: green fluorescent protein
gRNAs: guide RNAs
PAM: protospacer adjacent motif
