# Supplementary material for: Reconstitution of human CMG helicase ubiquitylation by CUL2LRR1 and multiple E2 enzymes
Source: Biochem J. 2021 Jul 23;478(14):2825–42. doi: 10.1042/BCJ20210315 (PMC8331092; doi:10.1042/BCJ20210315)
Supplement: Supplementary Figures S1-S4 and Table S1 [file BCJ-478-2825-s1.pdf]

Le et al, Figure S1

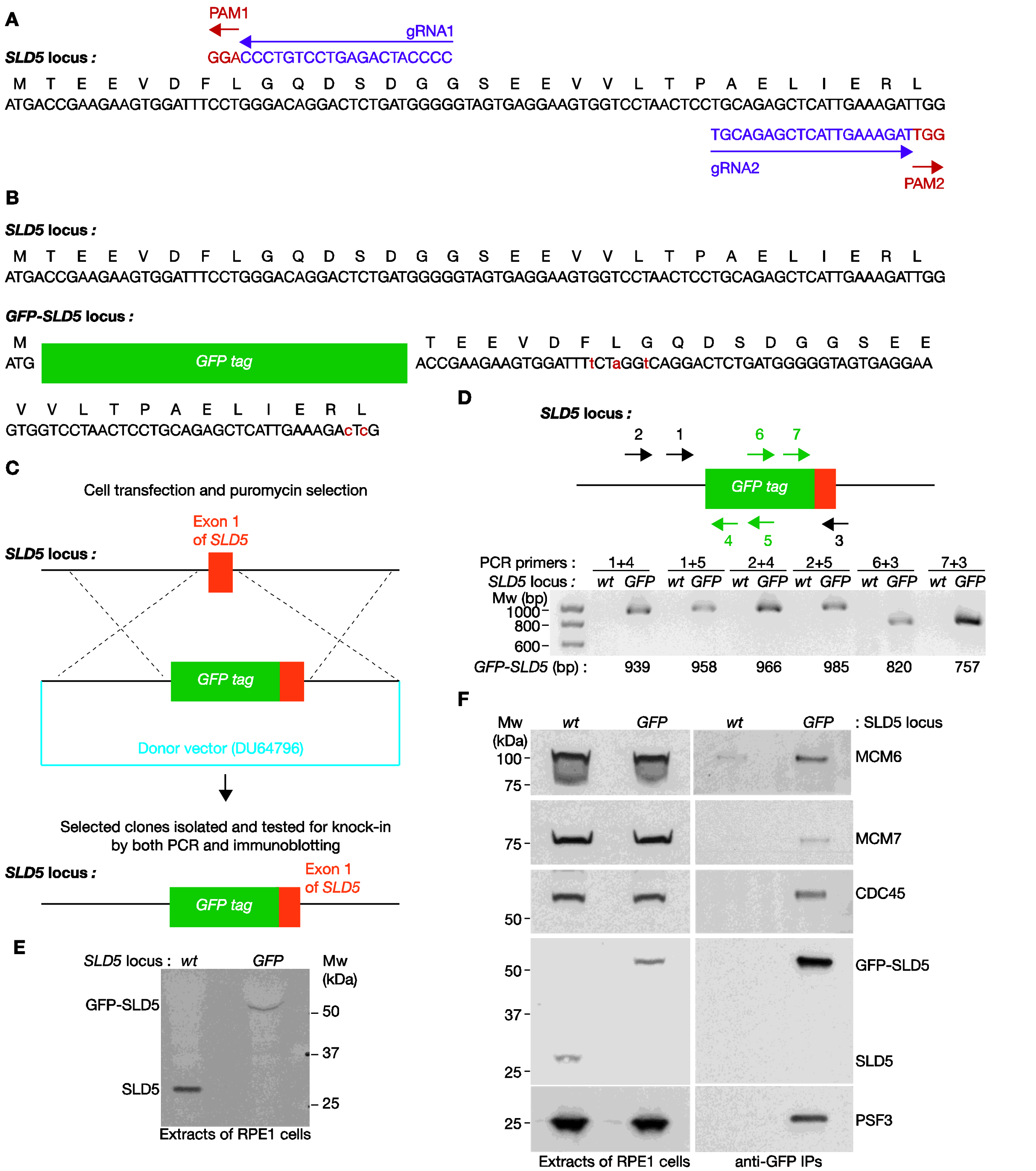

Using CRISPR-Cas9 to tag SLD5 with GFP in human RPE1 cells, as a tool for isolating human CMG.
(**A**) gRNAs used to target the 5' end of exon 1 of the *SLD5* gene in human RPE1 cells. Each gRNA contained 20-nt homology to a site in the genome that was immediately followed by a 3-nt "Protospacer Adjacent Motif" or PAM sequence that has the form "NGG" and is required for cleavage by Cas9.
(**B**) Comparison of the wild type *SLD5* locus with the *GFP-SLD5* locus generated by genome editing with CRISPR-Cas9. The donor DNA (see C below) contained silent mutations to inhibit cleavage by Cas9 after successful integration at the *SLD5* locus (highlighted in red and lowercase). (**C**) Scheme to introduce GFP at the beginning of exon 1 of *SLD5*. (**D**) PCR analysis of wild type and GFP-SLD5 with the indicated primers. (**E**) Immunoblot indicating successful tagging of both alleles of SLD5 with GFP. (**F**) The indicated cell lines, as confirmed by PCR analysis, subsequent DNA sequencing, and immunoblotting were used to isolated GFP-SLD5 on anti-GFP beads. Associated factors were monitored by immunoblotting as indicated.

Le et al, Figure S2

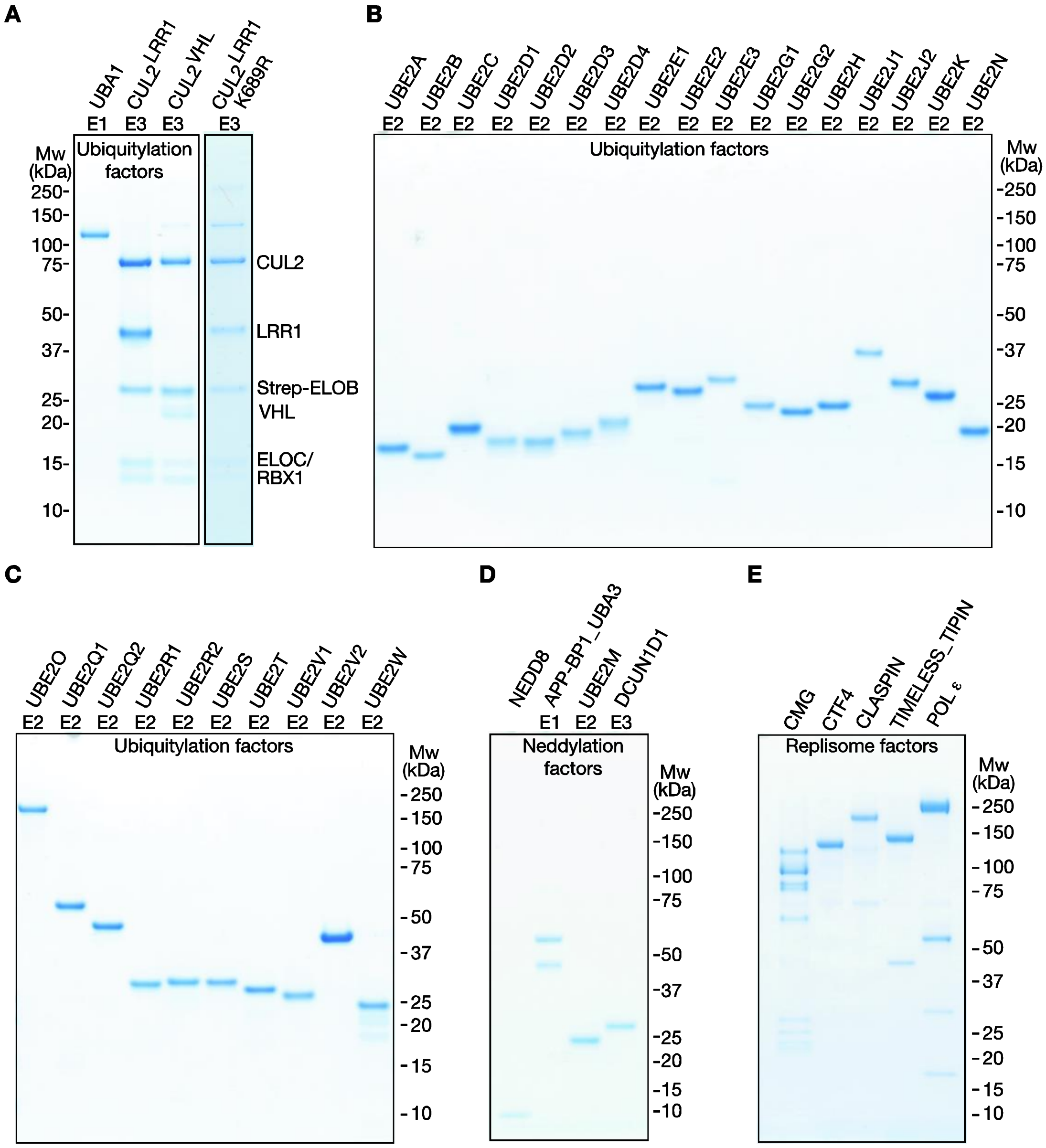

Purified proteins used in this study.
(**A**) UBA1, CUL2-LRR1 and CUL2-VHL were expressed in insect cells and purified as described in Materials and Methods.
(**B**-**D**) The indicated factors were purified and kindly provided by MRC PPU Reagents and Services and Ubiquigent (in the case of NEDD8).
(**E**) The indicated replisome proteins were expressed in budding yeast or insect cells and purified as described in Materials and Methods.
Further details of all the purified proteins in this figure are provided in Table S1, as well as in Materials and Methods.

Le et al, Figure S3

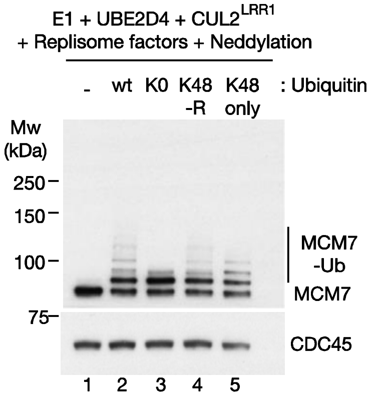

Polyubiquitylation of CMG-MCM7 by UBE2D4 is inefficient and is independent of lysine 48 of ubiquitin.
Reactions containing UBE2D4 were performed as in Figure 2A, but in the presence or absence of the indicated variants of ubiquitin.

Le et al, Figure S4

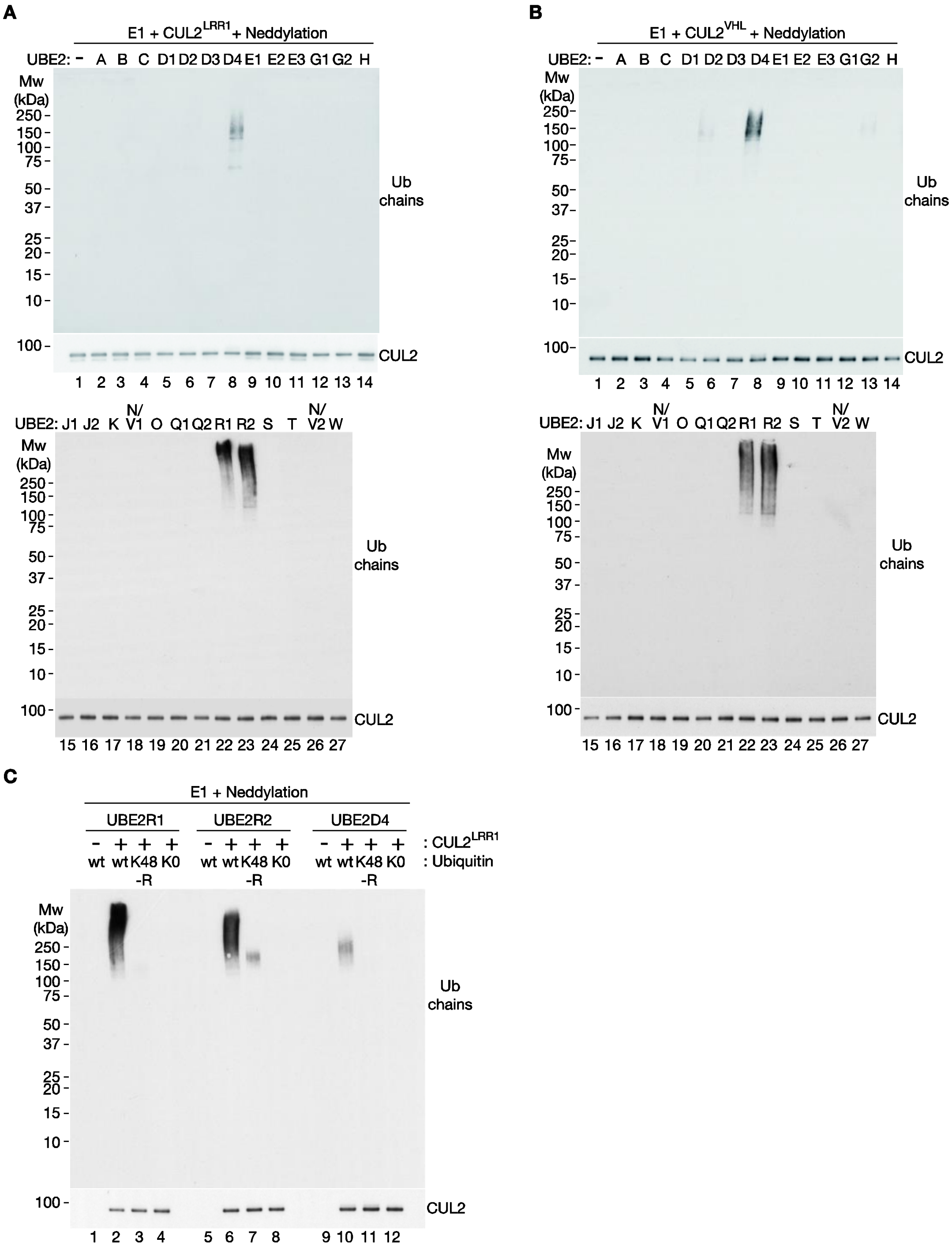

CUL2-LRR1 and CUL2-VHL stimulate the formation of ubiquitin chains by UBE2R1/R2 (and UBE2D4).
(**A**-**B**) Reactions were performed as in Figure 2A-B, but in the absence of CMG and other replisome factors. The formation of poly-ubiquitin chains was monitored by immunoblotting. 'Neddylation' indicates the presence of the factors shown in Figure S2D. (**C**) Similar reactions with the indicated E2 / E3 enzymes and the indicated variants of ubiquitin (as described above for Figure 4).

Table S1: Reagents and resources used in this study

**REAGENT or RESOURCE**	SOURCE	IDENTIFIER	Additional information
**Antibodies**			
CDC45 (antigen 1-222 mouse)<br>1000X dilution for immunoblotting	MRC PPU Reagents and Services	DU35753	Sheep polyclonal antibody
MCM2 (antigen 1-222 mouse)<br>5000X dilution for immunoblotting	MRC PPU Reagents and Services	DU24572	Sheep polyclonal antibody
MCM3 (antigen 1-222 mouse)<br>1000X dilution for immunoblotting	MRC PPU Reagents and Services	DU51847	Sheep polyclonal antibody
MCM4 (antigen 1-222 mouse)<br>3000X dilution for immunoblotting	MRC PPU Reagents and Services	DU51810	Sheep polyclonal antibody
MCM5 (antigen 1-222 mouse)<br>3000X dilution for immunoblotting	MRC PPU Reagents and Services	DU51792	Sheep polyclonal antibody
MCM6 (antigen 1-222 mouse)<br>3000X dilution for immunoblotting	MRC PPU Reagents and Services	DU51793	Sheep polyclonal antibody
MCM7 (antigen 1-719 human)<br>500X dilution for immunoblotting	Santa Cruz Biotechnology	sc-9966	Mouse monoclonal antibody
SLD5 (antigen 1-223 mouse)<br>1000X dilution for immunoblotting of purified protein<br>2000X dilution for immunoblotting of cell extract samples	MRC PPU Reagents and Services	DU24572	Sheep polyclonal antibody
PSF1 (antigen 1-196 mouse)<br>1000X dilution for immunoblotting of purified protein<br>500X dilution for immunoblotting of cell extract samples	MRC PPU Reagents and Services	DU51938	Sheep polyclonal antibody
PSF2 (antigen 1-185 mouse)<br>1000X dilution for immunoblotting of purified protein<br>2000X dilution for immunoblotting of cell extract samples	MRC PPU Reagents and Services	DU51832	Sheep polyclonal antibody
PSF3 (antigen 1-216 mouse)<br>1000X dilution for immunoblotting	MRC PPU Reagents and Services	DU24601	Sheep polyclonal antibody
CTF4 (antigen 781-1001 mouse)<br>1000X dilution for immunoblotting	MRC PPU Reagents and Services	DU56612	Sheep polyclonal antibody
CLASPIN (antigen 1-222 mouse)	MRC PPU	DU56412	Sheep polyclonal

1000X dilution for immunoblotting	Reagents and Services		antibody
TIMELESS (antigen 1-222 mouse)<br>1000X dilution for immunoblotting	MRC PPU Reagents and Services	DU56427	Sheep polyclonal antibody
TIPIN (antigen 1-222 mouse)<br>3000X dilution for immunoblotting	MRC PPU Reagents and Services	DU56426	Sheep polyclonal antibody
POLE1 (antigen 1-222 mouse)<br>1000X dilution for immunoblotting	MRC PPU Reagents and Services	DU27959	Sheep polyclonal antibody
POLE2 (antigen 1-222 mouse)<br>1000X dilution for immunoblotting	MRC PPU Reagents and Services	DU56428	Sheep polyclonal antibody
POLE4 (antigen 1-117 human)<br>1000X dilution for immunoblotting	MRC PPU Reagents and Services	DU 62488	Sheep polyclonal antibody
FK2<br>100X dilution for immunoblotting	Enzo Life Sciences	BML-PW8810-0100	Mouse monoclonal antibody
CUL2-RBX1<br>3000X dilution for immunoblotting	MRC PPU Reagents and Services	RA334	Rabbit polyclonal antibody
Streptavidin-HRP<br>500X dilution for immunoblotting	Millipore	18-152	N/A
Sheep IgG HRP<br>20000X dilution for immunoblotting	Sigma-Aldrich	A3415	Donkey polyclonal antibody
Mouse IgG HRP<br>5000X dilution for immunoblotting	Sigma-Aldrich	A4416	Goat polyclonal antibody
Rabbit IgG HRP<br>10000X dilution for immunoblotting	Sigma-Aldrich	A6667	Goat polyclonall antibody
**Peptides and Recombinant proteins**			
HIF1A Peptide Substrate, Biotin	R&D systems	S-340-010	N/A
HIF1A Negative Control Peptide, Biotin	R&D systems	S-345-010	N/A
UBA1	This study	N/A	Human
UBE2A	MRC PPU Reagents and Services	DU4203	Human
UBE2B	MRC PPU Reagents and Services	DU20017	Human
UBE2C	MRC PPU Reagents and Services	DU32146	Human
UBE2D1	MRC PPU	DU4315	Human

	Reagents and Services		
UBE2D2	MRC PPU Reagents and Services	DU20184	Human
UBE2D3	MRC PPU Reagents and Services	DU15703	Human
UBE2D4	MRC PPU Reagents and Services	U8232	Human
UBE2E1	MRC PPU Reagents and Services	DU12803	Human
UBE2E2	MRC PPU Reagents and Services	DU12394	Human
UBE2E3	MRC PPU Reagents and Services	DU14049	Human
UBE2G1	MRC PPU Reagents and Services	DU14055	Human
UBE2G2	MRC PPU Reagents and Services	SC20174	Human
UBE2H	MRC PPU Reagents and Services	DU32149	Human
UBE2J1 (amino acids 1-282)	MRC PPU Reagents and Services	DU20687	Human TM domain truncation
UBE2J2 (amino acids 1-226)	MRC PPU Reagents and Services	DU20695	Human TM domain truncation
UBE2K	MRC PPU Reagents and Services	SC20018	Human
UBE2N	MRC PPU Reagents and Services	DU15705	Human
UBE2O	MRC PPU Reagents and Services	DU45549	Human
UBE2Q1	MRC PPU Reagents and	DU20176	Human

	Services		
UBE2Q2	MRC PPU Reagents and Services	DU12801	Human
UBE2R1 / CDC34	This study	N/A	Human
UBE2R2	MRC PPU Reagents and Services	DU4616	Human
UBE2S	MRC PPU Reagents and Services	DU20175	Human
UBE2T	MRC PPU Reagents and Services	DU12416	Human
UBE2V1	MRC PPU Reagents and Services	DU20179	Human
UBE2V2	MRC PPU Reagents and Services	DU11887	Human
UBE2W	MRC PPU Reagents and Services	DU20190	Human
$CUL2^{LRR1}$	This study	N/A	Human
$CUL2^{LRR1}$ K689R	This study	N/A	Human
$CUL2^{VHL}$	This study	N/A	Human
Ubiquitin wildtype	Axel Knebel	DU20027	Human
Ubiquitin K0	Axel Knebel	DU24363	Human
Ubiquitin K48R	Axel Knebel	DU20042	Human
Ubiquitin K48-only	Axel Knebel	DU24367	Human
NEDD8	UBIQUIGENT	60-0009-500	Human
APP-BP1_UBA3	MRC PPU Reagents and Services	DU21784	Human<br>E1 for neddylation
UBE2M	MRC PPU Reagents and Services	DU15804	Human<br>E2 for neddylation
DCUN1D1	MRC PPU Reagents and Services	DU20631	Human<br>E3 for neddylation
CMG	This study	N/A	Human
CTF4	This study	N/A	Human
CLASPIN	This study	N/A	Human
TIMELESS_TIPIN	This study	N/A	Human

POL ε	This study	N/A	Human
TEV protease	Axel Knebel	N/A	N/A
Ulp1 protease	This study & (Stein, Ruggiano et al., 2014)	N/A	N/A
3FLAG peptide	Sigma-Aldrich	F4799	N/A
Propargyl- Ubiquitin	MRC PPU Reagents and Services	DU49003	N/A
USP2	MRC PPU Reagents and Services	DU32888	Human
**Chemicals, Reagents**			
Human XpressRef Universal Total RNA	QIAGEN	338112	N/A
PrimeScript™ RT-PCR Kit	Takara	RR014A	N/A
Anti-FLAG M2 affinity gel	Sigma-Aldrich	A2220	N/A
Ni-NTA agarose	Qiagen	30210	N/A
IgG Sepharose 6 Fast Flow	GE Healthcare	17096901	N/A
Strep-Tactin Superflow	IBA Lifesciences	2-1206-025	N/A
Sigma protease inhibitor cocktail	Sigma-Aldrich	P8215	N/A
Roche Complete EDTA-free protease inhibitor cocktail	Roche	11873580001	N/A
Sf-900™ II SFM	Gibco	10902104	N/A
Antibiotic-Antimycotic	Gibco	15240096	N/A
Cellfectin™ II Reagent	Gibco	10362100	N/A
Dulbecco's Modified Eagle Medium:Nutrion Mixture F- 12	Thermo Fisher	21331020	N/A
L-Glutamine	Thermo Fisher	25030081	N/A
Foetal Bovine Serum	LabTech	FCS-SA/500	N/A
Penicillin-Streptomycin	Thermo Fisher	15140122	N/A
Lipofectamine 2000	Thermo Fisher	11668030	N/A
0.05% Trypsin / EDTA	Thermo Fisher	25300054	N/A
Puromycin	Thermo Fisher	A1113802	N/A
CB-5083	Selleckchem	S8101	N/A
MLN-4924	Activebiochem	A1139	N/A
PrimeSTAR hot-start DNA polymerase	Takara Bio	R010B	N/A
Pierce Universal Nuclease	Thermo Fisher	88702	N/A
GFP-Trap agarose beads	Chromatek	Gta-100	N/A
***E. coli* strains**			
Rosetta (DE3) pLysS	Novagen	70956	N/A

Max Efficiency[TM] DH10Bac Competent Cells	Gibco	10361012	N/A
**Yeast strains**			
YCPR26<br>(*Saccharomyces cerevisiae*, strain background)<br>*MAT***a** / *MAT*α<br>*bar1Δ::HphNT / bar1Δ::HphNT*<br>*pep4Δ::kanMX / pep4Δ::kanMX*<br>*ade2-1 / ade2-1*<br>*leu2-3::pRS305-MCM4-MCM5 / leu2-3:: pRS305-PSF2-PSF3*<br>*ura3-1:: pRS306-MCM2-MCM3 / ura3-1::pRS306-PrA-3TEV-SLD5-PSF1*<br>*his3-11 / his3-11:: pRS303-CDC45*<br>*trp1-1::pRS304-MCM6-MCM7/ trp1-1*	This study	N/A	For CMG purification
yCPR4<br>(*Saccharomyces cerevisiae*, strain background)<br>*MAT***a**<br>*bar1Δ::HphNT*<br>*pep4Δ::kanMX*	This study	N/A	For CMG purification strain making
yJF1<br>(*Saccharomyces cerevisiae*, strain background)<br>*MAT***a**<br>*bar1Δ::HphNT*<br>*pep4Δ::kanMX*	(Frigola, Remus et al., 2013)	N/A	For CMG purification strain making
**Insect cell lines**			
Sf9	Invitrogen	11496015	N/A
Sf21	Invitrogen	11497013	N/A
**Mammalian cell lines**			
Human hTERT RPE1 (Retinal Pigment Epithelial) cells	N/A	N/A	Confirmed by 'Short Tandem Repeat profiling' via ATCC
*GFP-SLD5 (*human hTERT RPE1 cells)	This study	N/A	N/A
**Plasmid DNA**			
pLIB	(Weissmann, Petzold et al., 2016)	N/A	N/A
pBIG1a	(Weissmann et al., 2016)	N/A	N/A
pBIG1b	(Weissmann et	N/A	N/A

	al., 2016)		
pTL1 (pLIB-5XFLAG_Tev_UBA1)	This study	DU70578	For expression of UBA1 in insect cells
pTL2 (pK27Sumo-CDC34)	This study	DU70579	For expression of UBE2R1 / CDC34 in *E. coli* cells
pTL3 (pBIG1a-CUL2_RBX1)	This study	-DU70580	For expression of $CUL2^{LRR1}$ and $CUL2^{VHL}$ in insect cells
pTL4 (pBIG1a-CUL2K689R_RBX1)	This study	-DU70581	For expression of $CUL2^{LRR1}$ K689R in insect cells
pTL5 (pBIG1b-LRR1_Strep_Tev_ELOB_ELOC)	This study	-DU70582	For expression of $CUL2^{LRR1}$ and $CUL2^{LRR1}$ K689R in insect cells
pTL6 (pBIG1b-VHL_Strep_Tev_ELOB_ELOC)	This study	DU70583	For expression of $CUL2^{VHL}$ in insect cells
pTL7 (pLIB-Strep_Tev_CTF4)	This study	DU70584	For expression of CTF4 in insect cells
pTL8 (pLIB-Strep_Tev_CLSPN)	This study	DU70585	For expression of CLASPIN in insect cells
pTL9 (pLIB-Strep_Tev_TIMELESS)	This study	DU70586	For expression of TIMELESS-TIPIN in insect cells
pTL10 (pLIB-TIPIN)	This study	DU70587	For expression of TIMELESS-TIPIN in insect cells
pTL11 (pLIB-POLE1)	This study	DU70588	For expression of POLε in insect cells
pTL12 (pBIG1b-POLE2_Strep_Tev_POLE3_POLE4)	This study	DU70589	For expression of POLε in insect cells
pCPR1 (pRS303-CDC45-Gal1-10)	This study	DU70590	For CMG purification
pCPR2 (pRS304-MCM6-Gal1-10-MCM7)	This study	DU70591	For CMG purification
pCPR3 (pRS305-MCM4-Gal1-10-MCM5)	This study	DU70592	For CMG purification
pCPR4 (pRS305-PSF2-Gal1-10-PSF3)	This study	DU70593	For CMG purification
pCPR5 (pRS306-MCM2-Gal1-10-MCM3)	This study	DU70594	For CMG purification

pCPR6 (pRS303-PrA-3TEV-SLD5-Gal1-10-PSF1)	This study	DU70595	For CMG purification
pX335	Addgene (Pyzocha et al (2014))	42335	Expression vector for gRNA and Cas9-D10A ‘nickase’
pBABED P U6	MRC PPU Reagents and Services	DU48788	Expression vector for gRNA
gRNA1 SLD5 plasmid	MRC PPU Reagents and Services	DU64805	Target sequence inserted into pX335
gRNA2 SLD5 plasmid	MRC PPU Reagents and Services	DU64804	Target sequence inserted into pBABED P U6
Donor vector for GFP-SLD5 (N-terminal GFP-TEV-5xGA-SLD5 Knock-in)	MRC PPU Reagents and Services	DU64796	N/A
**DNA oligonucleotides**			
**8228** CCACCATCGGGCGCGGATCCA ATGTCCAGCTCGCCGCTGTCCA AGAAACG	This study	N/A	Forward primer for sub-cloning of *UBA1* into pLIB
**8229** TCCTCTAGTACTTCTCGACAAG CTTTCAGCGGATGGTGTATCGG ACATAGGGAAC	This study	N/A	Reverse primer for sub-cloning of *UBA1* into pLIB
**8277** gaacagattggtggcGCTCGGCCGCT AGTGCCCAGC	This study	N/A	Forward primer for sub-cloning of *UBE2R1* / *HsCDC34* into pK27SUMO
**8278** gtgcggccgcttattaGGACTCCTCCG TGCCAGAGTCATCCTC	This study	N/A	Reverse primer for sub-cloning of *UBE2R1* / *HsCDC34* into K27SUMO
**7873** CCACCATCGGGCGCGGATCCA ATGTCTTTGAAACCAAGAGTAG TAGATTTTG	This study	N/A	Forward primer for sub-cloning of *CUL2* into pLIB
**7874** TCCTCTAGTACTTCTCGACAAG CTTTCACGCGACGTAGCTGTAT TCATCTGC	This study	N/A	Reverse primer for sub-cloning of *CUL2* into pLIB

**7881** CCACCATCGGGCGCGGATCCA ATGAAGCTACACTGTGAGGTGG AGGTG	This study	N/A	Forward primer for sub-cloning of *LRR1* into pLIB
**7882** TCCTCTAGTACTTCTCGACAAG CTTTTACTTTAACATATCAGAGG AATTAACATAAC	This study	N/A	Reverse primer for sub-cloning of *LRR1* into pLIB
**7875** CCACCATCGGGCGCGGATCCA ATGGACGTGTTCCTCATGATCC GGCGCCAC	This study	N/A	Forward primer for sub-cloning of *ELOB* into pLIB
**7876** TCCTCTAGTACTTCTCGACAAG CTTTCACTGCACGGCTTGTTCA TTGGCACTG	This study	N/A	Reverse primer for sub-cloning of *ELOB* into pLIB
**7877** CCACCATCGGGCGCGGATCCA ATGGATGGAGAGGAGAAAACCT ATGGTG	This study	N/A	Forward primer for sub-cloning of *ELOC* into pLIB
**7878** TCCTCTAGTACTTCTCGACAAG CTTTTAACAATCTAAGAAGTTCG CAGCCATC	This study	N/A	Reverse primer for sub-cloning of *ELOC* into pLIB
**7879** CCACCATCGGGCGCGGATCCA ATGGCGGCAGCGATGGATGTG GATACC	This study	N/A	Forward primer for sub-cloning of *RBX1* into pLIB
**7880** TCCTCTAGTACTTCTCGACAAG CTTCTAGTGCCCATACTTTTGGA ATTCCCAC	This study	N/A	Reverse primer for sub-cloning of *RBX1* into pLIB
**7883** CCACCATCGGGCGCGGATCCA ATGCCCCGGAGGGCGGAGAAC TGGGAC	This study	N/A	Forward primer for sub-cloning of *VHL* into pLIB
**7884** TCCTCTAGTACTTCTCGACAAG CTTTCAATCTCCCATCCGTTGAT GTGCAATG	This study	N/A	Reverse primer for sub-cloning of *VHL* into pLIB
**7834** CCACCATCGGGCGCGGATCCA ATGGACTTGCACATGATGAACT GTGAAC	This study	N/A	Forward primer for sub-cloning of *TIMELESS* into pLIB
**7835** TCCTCTAGTACTTCTCGACAAG	This study	N/A	Reverse primer for sub-cloning of

CTTTCAGTCATCCTCATCATCCTCAATCTGG			*TIMELESS* into pLIB
**7836** CCACCATCGGGCGCGGATCCAATGCTAGAACCACAGGAGAATGGCGTG	This study	N/A	Forward primer for sub-cloning of *TIPIN* into pLIB
**7837** TCCTCTAGTACTTCTCGACAAGCTTTTATCTAGCTTCAGTAATATTTCTGGATGTAG	This study	N/A	Reverse primer for sub-cloning of *TIPIN* into pLIB
**7840** CCACCATCGGGCGCGGATCCAATGGCGCCGGAGCGGCTGCGGAGCCG	This study	N/A	Forward primer for sub-cloning of *POLE*2 into pLIB
**7841** TCCTCTAGTACTTCTCGACAAGCTTTCAAAAGCCTTGAAGTTTGCTATCTTCTAC	This study	N/A	Reverse primer for sub-cloning of *POLE2* into pLIB
**7842** CCACCATCGGGCGCGGATCCAATGGCGGAGAGGCCCGAGGACCTAAACCTG	This study	N/A	Forward primer for sub-cloning of *POLE3* into pLIB
**7843** TCCTCTAGTACTTCTCGACAAGCTTTCAGTTGTCTACTTCTTCCTCTTCATTC	This study	N/A	Reverse primer for sub-cloning of *POLE3* into pLIB
**7844** CCACCATCGGGCGCGGATCCAATGGCGGCGGCGGCGGCGGCAGGAAG	This study	N/A	Forward primer for sub-cloning of *POLE4* into pLIB
**7845** TCCTCTAGTACTTCTCGACAAGCTTTCAATCTAAAGTACCTTCCAGAAAAGC	This study	N/A	Reverse primer for sub-cloning of *POLE4* into pLIB
**8407** ccggtcACTAGTtcaaggaagtaattatctactttttacaacaaatataaaacaATGTTCG	This study	N/A	*CDC45* forward primer for construction of pCPR1
**8408** tcttagCCCGGGgcataaaggcattaaaagaggagcg	This study	N/A	*CDC45* reverse primer for construction of pCPR1
**8416** gatccgcaGCGGCCGCtcaaggaagtaattatctactttttacaacaaatataaaacaATGGCTG	This study	N/A	*MCM2* forward primer for construction of pCPR5

**8417** ttacgtGAGCTCgcataaaggcattaaaagaggagc	This study	N/A	*MCM2* reverse primer for construction of pCPR5
**8428** gtagctACTAGTtataaaacaATGGCTGGTACTGTTGTTTTGGATGACG	This study	N/A	*MCM3* forward primer for construction of pCPR5
**8429** cagctaCCCGGGgcataaaggcattaaaagaggagc	This study	N/A	*MCM3* reverse primer for construction of pCPR5
**8439** tacgatccGCGGCCGCtcaaggaagtaattatctactttttacaacaaatataaaacaATGTCCTC	This study	N/A	*MCM4* forward primer for construction of pCPR3
**8440** ttcgagGAGCTCgcataaaggcattaaaagaggagc	This study	N/A	*MCM4* reverse primer for construction of pCPR3
**8451** cggtacACTAGTtcaaggaagtaattatctactttttacaacaaatataaaacaATGTCCG	This study	N/A	*MCM5* forward primer for construction of pCPR3
**8452** tggcatCCCGGGgcataaaggcattaaaagaggagc	This study	N/A	*MCM5* reverse primer for construction of pCPR3
**8461** tgacgaGCGGCCGCtataaaacaATGGATTTGGCTGCTGCTGCTGAACCAGGTG	This study	N/A	*MCM6* forward primer for construction of pCPR2
**8462** acgtcaGAGCTCgcataaaggcattaaaagaggagc	This study	N/A	*MCM6* reverse primer for construction of pCPR2
**8472** cgtaggACTAGTtataaaacaATGGCTTTGAAGGACTACGCTTTGGAAAAG	This study	N/A	*MCM7* forward primer for construction of pCPR2
**8473** taacgtCCCGGGgcataaaggcattaaaagaggagc	This study	N/A	*MCM7* reverse primer for construction of pCPR2
**8482** acgtcgACTAGTtcaaggaagtaattatct	This study	N/A	*PSF1* forward primer for

acttttacaacaaatataaaacaATGTTCTG			construction of pCPR6
**8483** ggtactCCCGGGgcataaaggcattaaaagaggagc	This study	N/A	*PSF1* reverse primer for construction of pCPR6
**8487** atgccaACTAGTtataaaacaATGGACGCTGCTGAAGTTGAATTCTTGGCTG	This study	N/A	*PSF2* forward primer for construction of pCPR4
**8488** ggctaatCCCGGGgcataaaggcattaaaagaggagc	This study	N/A	*PSF2* reverse primer for construction of pCPR4
**8492** tactgccgGCGGCCGCtcaaggaagtaattatctactttttacaacaaatataaaacaATGTCCG	This study	N/A	*PSF3* forward primer for construction of pCPR4
**8493** tgaacgGAGCTCgcataaaggcattaaaagaggagc	This study	N/A	*PSF3* reverse primer for construction of pCPR4
**8498** gatcaggcGCGGCCGCtataaaacaATGGGTTCTCCACAAC	This study	N/A	*SLD5* forward primer for construction of pCPR6
**8500** cattcgGAGCTCgcataaaggcattaaaagaggagc	This study	N/A	*SLD5* reverse primer for construction of pCPR6
**TM16466** caccgCCCCATCAGAGTCCTGTCCC	This study	N/A	Forward oligo annealed with TM16467 for insertion as gRNA1 into pX335 to generate DU64805
**TM16467** aaacGGGACAGGACTCTGATGGGGC	This study	N/A	Reverse oligo annealed with TM16466 for insertion as gRNA1 into pX335 to generate DU64805
**TM16464** caccgTGCAGAGCTCATTGAAAGAT	This study	N/A	Forward oligo annealed with TM16465 for insertion as gRNA2

			into pBABED P U6 to generate DU64804
**TM16465** aaacATCTTTCAATGAGCTCTGCAC	This study	N/A	Reverse oligo annealed with TM16464 for insertion as gRNA2 into pBABED P U6 to generate DU64804
**9222** CCTTGTGGCTCTGGGAAATTAGC	This study	N/A	*SLD5* forward primer (oligo 1 in Figure S1D) for PCR check
**9223** ATCGCCGACTGGTCTAATGTCG	This study	N/A	*SLD5* forward primer (oligo 2 in Figure S1D) for PCR check
**9224** AGTTACTGGCACATGGGAAATATGCA	This study	N/A	*SLD5* reverse primer (oligo 3 in Figure S1D) for PCR check
**9391** CCTTGAAGAAGATGGTGCGC	This study	N/A	*eGFP* reverse primer (oligo 4 in Figure S1D) for PCR check
**9392** GTCTTGTAGTTGCCGTCGTC	This study	N/A	*eGFP* reverse primer (oligo 5 in Figure S1D) for PCR check
**9393** CGACAACCACTACCTGAGCAC	This study	N/A	*eGFP* reverse primer (oligo 6 in Figure S1D) for PCR check
**9394** CATGGTCCTGCTGGAGTTCGTG	This study	N/A	*eGFP* reverse primer (oligo 7 in Figure S1D) for PCR check
